# On quasi-linear reaction diffusion systems arising from compartmental SEIR models

**DOI:** 10.1007/s00030-024-00985-w

**Published:** 2024-08-06

**Authors:** Juan Yang, Jeff Morgan, Bao Quoc Tang

**Affiliations:** 1 School of Mathematics and Statistics, Lanzhou University, Lanzhou, 730000 China; 2 Institute of Mathematics and Scientific Computing, University of Graz, Heinrichstrasse 36, 8010 Graz, Austria; 3 Department of Mathematics, University of Houston, Houston, Texas 77204 USA

**Keywords:** Quasi-linear reaction-diffusion systems, Global existence, $L^p$-energy methods, Mass control, 35A01, 35K57, 35K59, 35Q92

## Abstract

The global existence and boundedness of solutions to quasi-linear reaction-diffusion systems are investigated. The system arises from compartmental models describing the spread of infectious diseases proposed in Viguerie et al. (Appl Math Lett 111:106617, 2021); Viguerie et al. (Comput Mech 66(5):1131–1152, 2020), where the diffusion rate is assumed to depend on the total population, leading to quasilinear diffusion with possible degeneracy. The mathematical analysis of this model has been addressed recently in Auricchio et al. (Math Methods Appl Sci 46:12529–12548, 2023) where it was essentially assumed that all sub-populations diffuse at the same rate, which yields a positive lower bound of the total population, thus removing the degeneracy. In this work, we remove this assumption completely and show the global existence and boundedness of solutions by exploiting a recently developed $L^p$-energy method. Our approach is applicable to a larger class of systems and is sufficiently robust to allow model variants and different boundary conditions.

## Introduction

### Mathematical analysis of quasilinear SEIRD models

Modelling the spatial spread of infectious disease is a classical topic and has recently attracted much more interest from the mathematical community due to the Covid-19 epidemic. Classical models include the susceptible-infected-removed (SIR) system and its variants, such as susceptible-exposed-infected-removed (SEIR) or susceptible-exposed-infected-removed-deceased (SEIRD). To account for spatial heterogeneity, a compartmental model was proposed in [31, 32] where the diffusion coefficients are highly heterogeneous and depend on the total population density, leading to a *quasi-linear* reaction-diffusion system. The simulations therein showed a strong qualitative agreement from the forecast using the model and collected data in Lombardy.

Let $\Omega \subset {\mathbb {R}}^N$, $N\ge 1$, be a bounded domain^1^ with smooth boundary $\partial \Omega$ such that $\Omega$ lies locally on one side of $\partial \Omega$. Let *s*(*x*, *t*), *e*(*x*, *t*), *i*(*x*, *t*), *r*(*x*, *t*), and *d*(*x*, *t*) denote the susceptible, exposed, infected, recovered, and deceased population densities, respectively, at spatial position $x\in \Omega$ and at time $t>0$, and let $n(x,t):=s(x,t)+e(x,t)+i(x,t)+r(x,t)$ be the total living population density. The proposed model in [31] reads as

1.1 $$\begin{aligned} {\left\{ \begin{array}{ll} \partial _t s= \alpha n - (1 - A_0/n) \beta _i si - (1 - A_0/n) \beta _e se - \mu s + \nabla \cdot (n \nu _s \nabla s)\\ \partial _t e= (1 - A_0/n) \beta _i si + (1 - A_0/n) \beta _e se - \sigma e - \phi _e e - \mu e + \nabla \cdot (n \nu _e \nabla e)\\ \partial _t i = \sigma e - \phi _d i - \phi _r i - \mu i + \nabla \cdot (n \nu _i \nabla i)\\ \partial _t r = \phi _r i + \phi _e e - \mu r + \nabla \cdot (n \nu _r \nabla r)\\ \partial _t d = \phi _d i , \end{array}\right. } \end{aligned}$$

where the positive diffusion coefficients $\nu _s, \nu _e, \nu _i, \nu _r$, the birth rate $\alpha$, the inverse of the incubation period $\sigma$, the asymptomatic recovery rate $\phi _e$, the infected recovery rate $\phi _r$, the infected mortality rate $\phi _d$, the asymptomatic contact rate $\beta _e$, the symptomatic contact rate $\beta _i$, and the general mortality rate $\mu$ are positive and may depend on time and space.

The mathematical analysis of (1.1) was first studied in [1]. One distinguishing characteristic of this system, in comparison with many other variants of SIR models in the literature, is that the diffusion of all species depends on the total population density *n*, which makes (1.1) a *quasilinear* reaction-diffusion system. This also brings possible degeneracy to the diffusion operators and makes the problem more challenging. The global existence and boundedness of solutions to (1.1) was shown in [1] under the following assumptions and modifications:

(i) all diffusion rates are the same,^2^ i.e. $\nu _s = \nu _e = \nu _i = \nu _r = \nu$,
(ii) the terms $(1-A_0/n)$ and $\phi _di$ are replaced by a non-singular function *A*(*n*), for instance $A(n) = (1-A_0/n)_+$, and $\phi _dni$, respectively.

The replacement of $(1-A_0/n)$ avoids the singularity when *n* gets close to zero. A closer examination later reveals that this is not necessary since one can estimate $(si)/n \le s$ and $(se)/n\le e$. The assumptions of the same diffusion rate and replacement of $\phi _di$ are, on the other hand, essential as they help to obtain a *key property* that the total population density *n* is bounded *pointwise* from below by a constant for all time, provided the initial density $n_0$ is also bounded from below. Indeed, summing the equations of *s*, *e*, *i*, and *r*, keeping in mind the aforementioned modifications, yields an equation for *n* of the form

$$\begin{aligned} \partial _tn - \nabla \cdot (\nu n\nabla n) = -\phi _din + (\alpha -\mu )n {\ge (\alpha -\mu )n-\phi _d n^2} \end{aligned}$$

from which the lower bound for *n* on finite time intervals follows from the lower bound of initial data. With this lower bound of *n*, the diffusion operators become non-degenerate and the analysis can be carried out in a standard way. This strategy used in [1] seems to break down when the diffusion rates are different, but it still applies when the term $-\phi _d i$ stays in place, since in this case

$$\begin{aligned} \partial _tn - \nabla \cdot (\nu n\nabla n) = -\phi _di + (\alpha -\mu )n \ge (\alpha -\mu -\phi _d)n. \end{aligned}$$

So *n* is bounded from below on finite time intervals if ${n(x,0)\ge \varrho >0}$ for all $x\in \Omega$.

In this paper, we set out to remove the assumptions (i) and (ii) above and show the global existence and boundedness of solutions to the original system (1.1). In fact, we show the results for a much larger class of quasilinear reaction-diffusion systems which contains (1.1) as a special case. Our key idea is to exploit a recently developed $L^p$-energy approach in e.g. [12, 23] which does not require any lower bound on *n* except for its natural nonnegativity. In the next subsection, we provide the general setting while the main results and ideas are presented in Sect. 1.3.

### Problem setting

Let $1\le N\in \mathbb {N}$, and $\Omega \subset \mathbb {R}^N$ be a bounded domain with Lipschitz boundary $\partial \Omega$. Let $2\le m\in \mathbb {N}$. In this paper, we study the global existence and boundedness of the following *quasi-linear reaction-diffusion system* of concentrations $u=(u_1, \ldots , u_m)$, for any $i\in \{1,\ldots , m\}$,

1.2 $$\begin{aligned} {\left\{ \begin{array}{ll} \partial _t u_i-\nabla \cdot (D_i(x,t)\Phi (u)\nabla u_i)=f_i(x,t,u), &{}x\in \Omega , t>0\\ (D_i(x,t)\Phi (u)\nabla u_i)\cdot \eta =0, &{}x\in \partial \Omega , t>0\\ u_i(x,0)=u_{i,0}(x), &{}x\in \Omega , \end{array}\right. } \end{aligned}$$

where $\eta$ is the unit outward normal vector on $\partial \Omega$, initial date $u_{i,0}$ are bounded and non-negative, the diffusion matrix $D_i$: $\Omega \times [0,\infty )\rightarrow \mathbb {R}^{N\times N}$ satisfies

1.3 $$\begin{aligned} \lambda |\xi |^2\le \xi ^\top D_i(x,t)\, \xi , \quad \forall (x,t)\in \Omega \times [0,\infty ), ~\forall \xi \in \mathbb {R}^N, ~\forall i=1,\ldots ,m \end{aligned}$$

for some $\lambda >0$, and for each $T>0$,

1.4 $$\begin{aligned} D_{i}\in L^{\infty }(\Omega \times (0,T); \mathbb {R}^{N\times N}), \quad \forall i=1,\ldots ,m, \end{aligned}$$

and $$\Phi (u)$$ satisfies:

1. $${\Phi : \mathbb {R}^m_+\rightarrow \mathbb {R}_+}$$ is continuous;
2. There is some $b\ge 0$ and $M>0$, such that
  $$\begin{aligned} \Phi (u)\ge M \,u_i^b, \quad \forall u \in {\mathbb {R}}_+^m \text { and } i=1,\ldots ,m; \end{aligned}$$
3. There exist $\pi >0$ and ${\widetilde{M}}>0$ such that
  $$\begin{aligned} \Phi (u) \le {\widetilde{M}} \left( 1+\sum _{i=1}^{m}u_i^{\pi }\right) , \quad \forall u\in {\mathbb {R}}_+^m. \end{aligned}$$

The nonlinearities $f_i(x, t, u): \Omega \times \mathbb {R}_+\times \mathbb {R}^m_+\rightarrow \mathbb {R}$ satisfy the following conditions:

1. For any $i=1,\ldots ,m$ and any $(x,t)\in \Omega \times \mathbb {R}_+$,  $f_i(x,t,\cdot ): \mathbb {R}^m\rightarrow \mathbb {R}$ is locally Lipschitz continuous uniformly in $(x,t)\in \Omega \times (0,T)$ for any $T>0$;
2. For any $i=1,\ldots ,m$ and any $(x,t)\in \Omega \times \mathbb {R}_+$,  $f_i(x,t,\cdot )$ is quasi-positive, i.e., $f_i(x,t,u)\ge 0$ for all $u\in \mathbb {R}^m_+$ with $u_i=0$ for all $i=1,\ldots ,m$;
3. There exists $c_1,\ldots ,c_m>0$ and $K_1,K_2\in \mathbb {R}$ such that
  $$\begin{aligned} \sum _{i=1}^{m}c_if_i(x,t,u)\le K_1\sum _{i=1}^{m}u_i+K_2, \quad \forall (x,t,u)\in \Omega \times \mathbb {R}_+\times \mathbb {R}^m_+; \end{aligned}$$
4. There exist $K_3>0, r>0$, and a lower triangular matrix $A=(a_{ij})$ with positive diagonal entries, and nonnegative entries otherwise, such that, for any $i=1,\ldots ,m$
  $$\begin{aligned} \sum ^i_{j=1}a_{ij}f_j(x,t,u)\le K_3 \left( 1+\sum _{i=1}^{m}u_i^r\right) , \quad \forall (x,t,u)\in \Omega \times \mathbb {R}_+\times \mathbb {R}^m_+ \end{aligned}$$
  (we call this assumption an *intermediate sum of order* *r*);
5. The nonlinearities are bounded by a polynomial, i.e., there exists $l>0$ and $K_4>0$ such that $\forall i=1,\ldots , m$,
  $$\begin{aligned} |f_i(x,t,u)|\le K_4 \left( 1+\sum _{i=1}^{m}u_i^l\right) , \quad \forall (x,t,u)\in \Omega \times \mathbb {R}_+\times \mathbb {R}^m_+. \end{aligned}$$

The local Lipschitz continuity (A1) of the nonlinearities implies the existence of a local solution to (1.2) on a maximal interval $[0, T_{\max })$. The quasi-positivity assumption (A2) assures that the solution to (1.2) is non-negative (as long as it exists) if the initial data is non-negative, which is a natural assumption since we consider here $u_i$ as concentrations or densities. The assumption (A3) gives an upper bound on the total mass of the system. Reaction-diffusion systems satisfying (A1), (A2) and (A3) appear naturally in modeling many real life phenomena, ranging from chemistry, biology, ecology, or social sciences. Remarkably, these natural assumptions are not enough to ensure global existence of bounded solutions as it was pointed out by counterexamples in [26] and [27] even for *semilinear* systems, i.e. $\Phi (u)\equiv 1$ and $D_i(x,t)\equiv D_i$ in (1.2) where $D_i\ne D_j$ for some $i\ne j$. The study of global existence of semilinear systems, i.e. (1.2) with $\Phi (u)\equiv 1$, has made considerable progress in the last decade, see e.g. [4, 8, 10–12, 15, 17] and the survey [25].

One can readily check that all assumptions (Q1)–(Q3), (A1)–(A5) are fulfilled for the SEIR system (1.1) (excluding the equation of *d* as it is uncoupled) provided that the diffusion rates are bounded from below by positive constants, and all other rates are nonnegative and bounded functions of *x* and *t*. Indeed, by writing $u_1 = s$, $u_2 = e$, $u_3 = i$, $u_4 = r$, and $u = (u_1,\ldots ,u_4)$, we have $\Phi (u) = u_1+u_2+u_3 + u_4$ satisfying (Q1)–(Q3) with $b = \pi = 1$. Now (A1)–(A3) and (A5) are obviously fulfilled. To check (A4), we observe the first nonlinearity

$$\begin{aligned}&\alpha n - (1 - A_0/n) \beta _i si - (1 - A_0/n) \beta _e se - \mu s\\ {}&\qquad = \alpha n - \beta _i si + A_0\beta _i\frac{si}{n} - \beta _ese + A_0\beta _e \frac{se}{n} - \mu s\\&\qquad \le \alpha n + (A_0\beta _i + A_0\beta _e)s \end{aligned}$$

where we used $si/n \le s$ and $se/n\le e$ and the nonnegativity of the rates. Thanks to the boundedness of the rates, we see that the first nonlinearity is bounded above by a linear combination of all components. It’s immediate that the third and forth nonlinearities, as well as the sum of the first and second nonlinearities are bounded by a linear combination of all components, since all nonlinear terms are cancelled out when summing. Hence, by choosing $r = 1$ and the matrix

$$\begin{aligned} A = \begin{pmatrix} 1 &{} 0 &{} 0 &{} 0\\ 1 &{} 1 &{} 0 &{} 0\\ 0 &{} 0 &{} 1 &{} 0\\ 0 &{} 0 &{} 0 &{} 1 \end{pmatrix} \end{aligned}$$

we see that (A4) is satisfied. It is noted that all these assumptions also hold if we replace $-\phi _di$ in (1.1) by $-\phi _d in$ as done in [1]. Therefore, (1.1), as well as the modified one in [1], is indeed a special case of the general system (1.2).

### Main results

Let us start with the first main result about the global existence and boundedness of system (1.2).

#### Theorem 1.1

Assume (1.3), (1.4), (Q1), (Q2), (Q3), (A1), (A2), (A3), (A5) and (A4) with

1.5 $$\begin{aligned} 1\le r<1+b+\frac{2}{N}. \end{aligned}$$

Then for any nonnegative, bounded initial datum $u_0\in (L^{\infty }(\Omega ))^m$, there exists a global weak solution to (1.2) with $u_i\in L^{\infty }_{loc}(0,\infty ; L^{\infty }(\Omega ))$ for all $i=1,\ldots ,m.$ In particular, if $K_1<0$ or $K_1=K_2=0$, then the solution is bounded uniformly in time, i.e.

1.6 $$\begin{aligned} \sup _{t\ge 0}\Vert u_i(t)\Vert _{L^{\infty }(\Omega )}<+\infty , \quad \forall i=1,\ldots ,m. \end{aligned}$$

#### Remark 1.2

- When $b=0$, we have $\Phi (u)\ge M$. By letting $\Phi (u)=1$, our results cover the result in [12, Theorem 1.1].
- It is noted that under the assumption $K_1<0$ or $K_1 = K_2 = 0$, we can get the $L^1(\Omega )$-norm of the solution bounded uniformly in time (see Lemma 3.1). This fact can be used to show the uniform-in-time bound (1.6). As a result, if we can use other ideas or use other structure of the system to get the $L^1(\Omega )$-norm of the solution bound uniformly in time, we can remove the assumption $K_1<0$ or $K_1 = K_2 = 0$.
- It is noted that the key idea to prove global existence is to construct the $L^p$-energy function. Actually, the intermediate sum condition (A4) is essential to construct the $L^p$-energy function.

The proof of Theorem 1.1 is based on an $L^p$-energy approach. The traditional idea has been to construct an energy function that is decreasing or at least bounded in time for the (1.2) of the form

$$\begin{aligned} \mathscr {L}[u] = \sum _{i=1}^{m}\int _{\Omega }h_i(u_i) \,dx. \end{aligned}$$

It is noted that if $h_i(z) \sim z^p$, this yields an $L^p$-estimate of the solution, and for *p* large enough, using a bootstrapping procedure, one obtains an $L^\infty$-estimate which implies global existence. Unfortunately, this approach is very likely to fail under the general assumptions (A3)–(A4), except for some very special cases. The *duality method* (see e.g. [4, 13, 19, 20, 22, 25]) is very efficient when dealing with systems with *constant or smooth diffusion coefficients*. Using this method, one gets an $L^{2+\varepsilon }(\Omega \times (0,T))$-estimate from the mass control condition (A3). Combining this initial estimate, another duality argument (see [22]) and bootstrap argument, using the intermediate sum condition (A4) and the growth assumption (A5), one can obtain an $L^\infty (\Omega \times (0, T))$-estimate that ensures global existence (more details can be found in [22]). It is noted that this method does not extend to the case of merely bounded measurable or quasi-linear diffusion coefficients, unless some additional regularity assumptions are given (see [2, 6]). Recently, an $L^p$-energy approach was given in [12, 23], whose preliminary ideas have been used previously in, e.g., [14, 24]. It is remarked that a similar method has also been successfully applied to cross-diffusion systems, see [16]. The core idea of this $L^p$-energy method is to construct a generalized $L^p$-energy function of the following form:

$$\begin{aligned} \mathscr {L}_p[u](t)=\int _{\Omega } \mathscr {H}_p[u](t) \,d x, \end{aligned}$$

where $p\in \mathbb {N}$ with $p\ge 2$ and

$$\begin{aligned} \mathscr {H}_p[u](t)=\sum _{\beta \in \mathbb {Z}_{+}^m,|\beta |=p}\begin{pmatrix}p \\ \beta \end{pmatrix} \theta ^{\beta ^2} u(t)^\beta \end{aligned}$$

with

$$\begin{aligned} u(t)^\beta = \prod _{i=1}^{m}u_i^{\beta _i}, \theta ^{\beta ^2}= \prod _{i=1}^{m}\theta _i^{\beta ^2_i} \text { and } \begin{pmatrix}p \\ \beta \end{pmatrix}=\frac{p !}{\beta _{1} ! \cdots \beta _{m} !}, \end{aligned}$$

where $\theta =\left( \theta _1, \ldots , \theta _m\right)$ and $\theta _1, \ldots , \theta _m$ are positive real numbers which will be determined later. The function ${\mathscr {L}}_p[u]$ contains all (mixed) multi-variable polynomials of order *p* with carefully chosen coefficients. Thanks to the non-negativity of the solution, $(\mathscr {L}_p[u])^{1/p}$ is an equivalent $L^p$-estimate of *u*. Certainly, the difficulty is to choose these coefficients so that they are compatible with both diffusion and reactions. In our case, the assumptions on the quasilinear diffusion (Q2) and the intermediate sum condition (A4) allow us construct such an energy function.

Comparing to [12, 22], this work extends this $L^p$-energy method to the case of *degenerate quasi-linear diffusion coefficients*. Moreover, it is also shown that this method is sufficiently robust to model variants and different boundary conditions.

Conditionally, if we can, by using a specific structure, get a better a prior estimate of solutions, the exponent *r* in the intermediate sum condition (A4) can be enlarged. This is contained in the following theorem.

#### Theorem 1.3

Assume (1.3), (1.4), (Q1), (Q2), (Q3), (A1), (A2), (A4) and (A5). Suppose that there exists a constant $a\ge 1$ and $\mathscr {F}(T)\in (0, \infty )$ such that

1.7 $$\begin{aligned} \Vert u_i\Vert _{L^{\infty }(0,T;L^{a}(\Omega ))}\le \mathscr {F}(T), \quad \forall i=1,\ldots ,m, \, {\forall T\in (0, \infty )} \end{aligned}$$

and

1.8 $$\begin{aligned} 1\le r<1+b+\frac{2a}{N}. \end{aligned}$$

Then for any nonnegative, bounded initial datum $u_0\in (L^{\infty }(\Omega ))^m$, there exists a global weak solution to (1.2) with $u_i\in L^{\infty }_{loc}(0,\infty ; L^{\infty }(\Omega ))$ for all $i=1,\ldots ,m.$ In particular, if $\sup _{T\ge 0}\mathscr {F}(T)<\infty$, then the solution is bounded uniformly in time, i.e.

1.9 $$\begin{aligned} \sup _{t\ge 0}\Vert u_i(t)\Vert _{L^{\infty }(\Omega )}<+\infty , \quad \forall i=1,\ldots ,m. \end{aligned}$$

#### Remark 1.4

When $b=0$, our results cover the result in [12, Theorem 1.3].

#### Theorem 1.5

Assume (1.3), (1.4), (Q1), (Q2), (Q3), (A1), (A2), (A4) and (A5). Suppose that there exists a constant $q> 1$ such that

1.10 $$\begin{aligned} \Vert u_i\Vert _{L^{q}(0,T;L^{q}(\Omega ))}\le \mathscr {F}(T), \quad {\forall i=1,\ldots ,m}, \, {\forall T\in (0, \infty )} \end{aligned}$$

and

1.11 $$\begin{aligned} 1\le r< 1 + \frac{N}{N+2}b + \frac{2q}{N+2}. \end{aligned}$$

Then for any nonnegative, bounded initial datum $u_0\in (L^{\infty }(\Omega ))^m$, there exists a global weak solution to (1.2) with $u_i\in L^{\infty }_{loc}(0,\infty ; L^{\infty }(\Omega ))$ for all $i=1,\ldots ,m.$ In particular, if $\sup _{T\ge 0}\mathscr {F}(T)<\infty$, then the solution is bounded uniformly in time, i.e.

1.12 $$\begin{aligned} \sup _{t\ge 0}\Vert u_i(t)\Vert _{L^{\infty }(\Omega )}<+\infty , \quad \forall i=1,\ldots ,m. \end{aligned}$$

#### Remark 1.6

When $b=0$, our results cover the result in [12, Theorem 1.3].

It is worth noting that other conditions can also lead to a prior estimates. For example, the entropy condition has been considered in many papers, especially when it involves chemical reactions, see e.g. [3, 5, 29, 30]. This condition means that there exist the scalars $\mu _i\in \mathbb {R}$ such that

$$\begin{aligned} \sum _{i=1}^{m}f_i(x,t,u)(\log u_i+\mu _i)\le 0, \quad \forall (x,t,u)\in \Omega \times \mathbb {R}_+\times \mathbb {R}^m_+. \end{aligned}$$

This condition guarantees an $L^{1}(\Omega )$-estimate for $u_i\log u_i$. Actually, this condition guarantees an $L^{1}(\Omega )$-estimate for $H(u(\cdot ,t))$, where

$$\begin{aligned} H(u)=\sum _{i=1}^{m}u_i(\log u_i-1+\mu _i), \quad \forall u_i\ge 0. \end{aligned}$$

Moreover, we can assume there exists a set $M=\prod _{k=1}^m(\alpha _i,\beta _i)$, where $\alpha _i,\beta _i$ are extended real numbers such that $\alpha _i<\beta _i$ for each $i=1,\ldots , m$, and solutions to (1.2) remain in *M* if initial data lies in *M*, a function $H:M\rightarrow \mathbb {R}_+$ that is $C^2$ and has the form

$$\begin{aligned} H(u)=\sum _{i=1}^{m}h_i(u_i), \end{aligned}$$

where $$h_i:(\alpha _i,\beta _i)\rightarrow \mathbb {R}_+$$ satisfies

H1 $$\begin{aligned}&h_i^{\prime \prime }(z) \ge 0, \quad \forall z\in (\alpha _i,\beta _i);\\&\qquad h_i(z) \text { is bounded implies } z \text { is bounded};\\&\qquad \nabla H(u)\cdot {f}(x,t,u)\le K_5\sum _{i=1}^{m}h_i(u_i)\\ {}&\qquad +K_6, \quad \forall (x,t,u) \in \Omega \times \mathbb {R}_+\times \mathbb {R}_+^m \end{aligned}$$

for some $K_5, K_6>0$. Then, we would obtain an $L^1(\Omega )$-estimate for $H(u(\cdot ,t))$ (this has been introduced in [19]). In addition, intermediate sum conditions could also be written in the form

H2 $$\begin{aligned} A\begin{pmatrix}h_1'(u_1) {f}_1(x,t,u)\\ \vdots \\ h_m'(u_1) {f}_m(x,t,u)\end{pmatrix}\le K_7\textbf{1}\left( \sum _{i=1}^{m}h_i(u_i)+1\right) ^r, \quad \forall (x,t,u)\in \Omega \times \mathbb {R}_+\times \mathbb {R}_+^m, \end{aligned}$$

that would lead to results in the same manner as we obtain from (A3) and (A4) above.

#### Theorem 1.7

Assume (1.3), (1.4), (Q1), (Q2), (Q3), (A1), (A2), (A5), (H1) and (H2). Assume moreover that

1.13 $$\begin{aligned} 1\le r<1+b+\frac{2}{N}. \end{aligned}$$

Then for any nonnegative, bounded initial datum $u_0\in (L^{\infty }(\Omega ))^m$, there exists a global weak solution to (1.2) with $u_i\in L^{\infty }_{loc}(0,\infty ; L^{\infty }(\Omega ))$ for all $i=1,\ldots ,m.$ In particular, if $K_5<0$ or $K_5=K_6=0$, then the solution is bounded uniformly in time, i.e.

1.14 $$\begin{aligned} \sup _{t\ge 0}\Vert u_i(t)\Vert _{L^{\infty }(\Omega )}<+\infty , \quad \forall i=1,\ldots ,m. \end{aligned}$$

#### Remark 1.8

When $b=0$, our results cover the result in [12, Theorem 1.2].

Our method is sufficiently robust to extend to other boundary conditions (for example, Robin-type boundary conditions). The precise results are given in the following theorem.

#### Theorem 1.9

Consider the system

1.15 $$\begin{aligned} {\left\{ \begin{array}{ll} \partial _t u_i - \nabla \cdot (D_i(x,t)\Phi (u)\nabla u_i) = f_i(x,t,u), &{}x\in \Omega , t>0,\\ D_i(x,t)\Phi (u)\nabla u_i(x,t)\cdot \eta + \alpha _i u_i(x,t) = 0, &{}x\in \partial \Omega , t>0,\\ u_{i}(x,0) = u_{i,0}(x), &{}x\in \Omega , \end{array}\right. } \end{aligned}$$

where $\eta$ is the unit outward normal vector on $\partial \Omega$, and $\alpha _i\ge 0$ for all $i=1,\ldots , m$.

Assume (1.3), (1.4), (Q1), (Q2), (Q3), (A1), (A2), (A4), (A5). Moreover, assume either (A3) or (1.7) or (1.10) with

$$\begin{aligned} 0\le r< {\left\{ \begin{array}{ll} 1 + b + \frac{2}{N}, &{}\text { in case of } (A3),\\ 1 + b + \frac{2a}{N}, &{}\text { in case of } (1.7),\\ 1 + \frac{N}{N+2}b + \frac{2q}{N+2}, &{}\text { in case of } (1.10). \end{array}\right. } \end{aligned}$$

Then for any non-negative, bounded initial data $u_0\in L^{\infty }(\Omega )^m$, there exists a global weak solution to (1.15) (see Definition 4.1) with $u_i\in L^{\infty }_{loc}(0,\infty ;L^{\infty }(\Omega ))$ for all $i=1,\ldots , m$. In particular, if $K_1< 0$ or $K_1= K_2 = 0$ in case of (A3), or $\sup _{T\ge 0}\mathscr {F}(T)<\infty$ in case of (1.7) or (1.10), then the solution is bounded uniformly in time, i.e.

$$\begin{aligned} \sup _{t\ge 0}\Vert u_i(t)\Vert _{L^{\infty }(\Omega )}<+\infty , \quad \forall i=1,\ldots ,m. \end{aligned}$$

#### Remark 1.10

We believe Theorem 1.9 can also be extended to the semilinear boundary conditions of the form

$$\begin{aligned} D_i(x,t)\nabla u_i(x,t)\cdot \eta + \alpha _i u_i(x,t) = G_i(u), \quad x\in \partial \Omega , \end{aligned}$$

where the nonlinearities $G_i$ also satisfy a quasi-positivity condition and an intermediate sum condition. The details are left for the interested reader. We refer to [23, 28] for a related work dealing with constant diffusion coefficients.

**The paper is organised as follows.** In Sect. 2, we prove the existence of Theorem 1.1 by first considering an approximate system where we regularize the nonlinearities to obtain global approximate weak solutions. Then we derive uniform estimates, applying the key idea of the $L^p$-energy functions, and pass to the limit to obtain global existence of (1.2). The uniform-in-time boundedness is proved in Sect. 3. The proofs of extended Theorems 1.3, 1.5, 1.7, and 1.9 are given in Sect. 4. In Sect. 5, we show application of our results to a Susceptible-Exposed-Infected-Recovered (SEIRD) model and its variants. Finally, in Appendix A, we give the proof of Lemma 2.9.

**Notation.** In this paper we use the following notation, some of which will be recalled from time to time:

- For $T>0$ and $p\in [1,\infty ]$, $Q_T:= \Omega \times (0,T)$ and
  $$\begin{aligned} L^p(Q_T):= L^p(0,T;L^p(\Omega )) \end{aligned}$$
  equipped with the usual norm
  $$\begin{aligned} \Vert f\Vert _{L^{p}(Q_T)}:= \left( \int _0^T\int _{\Omega }|f|^pdxdt\right) ^{1/p} \end{aligned}$$
  for $$1\le p < \infty $$ and
  $$\begin{aligned} \Vert f\Vert _{L^{\infty }(Q_T)}:= \underset{(x,t)\in Q_T}{\text { ess sup}}|f(x,t)|. \end{aligned}$$
- For $p\in [1,\infty ]$, $\tau \ge 0$ and $\delta >0$, we denote by
  $$\begin{aligned} Q_{\tau ,\tau +\delta }:= \Omega \times (\tau ,\tau +\delta ) \end{aligned}$$
  and
  $$\begin{aligned} L^p(Q_{\tau ,\tau +\delta }):= L^p(\tau ,\tau +\delta ; L^p(\Omega )). \end{aligned}$$

## Global existence of bounded weak solutions

### Definition 2.1

(Weak solutions) A vector of nonnegative state variables $u=(u_1,\ldots ,u_m)$ is called a weak solution to (1.2) on (0, *T*) if

$$\begin{aligned}{} & {} \partial _tu_i\in L^2(0,T;(H^1(\Omega ))^{\prime }), \quad \Phi (u)\nabla u_i\in L^2(0,T; L^2(\Omega )^N),\\{} & {} \quad {u_i \in C(0,T; L^2(\Omega )), \quad u_i^{\frac{b+2}{2}} \in L^2(0,T; H^1(\Omega )) }, \end{aligned}$$

where *b* is in (Q2), and

$$\begin{aligned} {f_i(u) \in L^2(0,T; L^2(\Omega ))} \end{aligned}$$

with $u_i(\cdot , 0)=u_{i,0}(\cdot )$ for all $i=1,\ldots ,m$, and for any test function ${\psi } \in L^2(0,T; H^1(\Omega ))$ we have

$$\begin{aligned} \begin{aligned} \int ^T_0 \int _{\Omega }\partial _t u_i \psi \,dxdt+\int ^T_0 \int _{\Omega }D_i(x,t)\Phi (u)\nabla u_i \cdot \nabla \psi \,dxdt =\int ^T_0 \int _{\Omega }f_i(u)\psi \,dxdt. \end{aligned} \end{aligned}$$

### Approximate system

For $i=1,\ldots ,m$ and $\varepsilon >0$, consider the following approximating system for $u^{\varepsilon }=(u_1^{\varepsilon }, \ldots , u_m^{\varepsilon })$,

2.1 $$\begin{aligned} {\left\{ \begin{array}{ll} \partial _t u_i^{\varepsilon }-\nabla \cdot (D_i(x,t)\Phi (u^{\varepsilon })\nabla u_i^{\varepsilon })=f^{\varepsilon }_i(u^{\varepsilon }), &{}x\in \Omega , t>0,\\ (D_i(x,t) \Phi (u^{\varepsilon }) \nabla u^{\varepsilon }_i)\cdot \eta =0, &{}x\in \partial \Omega , t>0,\\ u^{\varepsilon }_i(x,0)=u_{i,0}(x)+\varepsilon , &{}x\in \Omega , \end{array}\right. } \end{aligned}$$

where

$$\begin{aligned} f^{\varepsilon }_i(u^{\varepsilon }):=\frac{f_i(u^{\varepsilon })}{1+\varepsilon \sum ^m_{j=1}|f_j(u^{\varepsilon })|} \end{aligned}$$

and $u_{i,0}^{\varepsilon }\in L^{\infty }(\Omega )$ is non-negative and $u_{i,0}^{\varepsilon }{\mathop {\longrightarrow }\limits ^{\varepsilon \rightarrow 0}}u_{i,0}$ in $L^{\infty }(\Omega )$. With this approximation, it is easy to check that the approximated non-linearities $f_i^{\varepsilon }$ still satisfy the assumptions (A1)–(A5).

#### Lemma 2.2

For any fixed $\varepsilon >0$, there exists a global bounded, nonnegative weak solution to (2.1) on any finite time interval (0, *T*), $T>0$.

#### Proof

Since for fixed $\varepsilon >0$, the nonlinearities $f_i^{\varepsilon }(u^{\varepsilon })$ are Lipschitz continuous and bounded, i.e.,

$$\begin{aligned} f^{\varepsilon }_i(u^{\varepsilon }):=\frac{f_i(u^{\varepsilon })}{1+\varepsilon \sum ^m_{j=1}|f_j(u^{\varepsilon })|}\le \frac{1}{\varepsilon }, \quad \forall (x,t)\in Q_T. \end{aligned}$$

The global existence of a weak solution of (2.1) is standard (see e.g. [18, Chapter VII]).

Next we prove the nonnegativity of $u^{\varepsilon }$. Denote $u_{i,+}^{\varepsilon }:=\max \{u_i^{\varepsilon },0\}$ and $u_{i,-}^{\varepsilon }:=\min \{u_i^{\varepsilon },0\}$. We consider the auxiliary system of (2.1)

$$\begin{aligned} \partial _t u_i^{\varepsilon }-\nabla \cdot (D_i(x,t)\Phi (u^{\varepsilon })\nabla u_i^{\varepsilon })=f^{\varepsilon }_i(u_+^{\varepsilon }), \end{aligned}$$

where $$u_+^{\varepsilon }=(u_{i,+}^{\varepsilon })_{i=1,\ldots ,m}$$.

By multiplying this auxiliary system by $u_{i,-}^{\varepsilon }$ and using the quasi-positivity assumption (A2) (recall that this property also holds for $f_i^{\varepsilon }$), we obtain

$$\begin{aligned} \frac{1}{2}\int _{\Omega }|u_{i,-}^{\varepsilon }|^2 \,dx+\lambda \int _{\Omega }\Phi (u^{\varepsilon }) |\nabla u_{i,-}^{\varepsilon }|^2\,dx \le 0, \end{aligned}$$

where we use $u_{i,0,-}^{\varepsilon }=0$. Thus, we get $u_{i,-}^{\varepsilon }=0$ a.e. in $Q_T$, this shows the desired nonnegativity. $\square$

### Uniform-in-$\varepsilon$ estimate

In this subsection, we prove crucial uniform-in-$\varepsilon$ estimates for the solution to (2.1). Moreover, we want to emphasize that all the constants in this subsection are independent of $\varepsilon$.

The following bound in $L^{\infty }(0,T; L^1(\Omega ))$ is immediate.

#### Lemma 2.3

Assume (A1), (A2) and (A3). Then for any $T>0$, there exists a constant $M_T$ depending on $T, \Omega , \Vert u_{i,0}\Vert _{L^1(\Omega )}$ and $c_1,\ldots ,c_m$, $K_1, K_2$ in (A3) such that

$$\begin{aligned} \sup _{t\in (0,T)}\Vert u_i^{\varepsilon }(t)\Vert _{L^{1}(\Omega )}\le M_T, \quad \forall i=1,\ldots ,m. \end{aligned}$$

#### Proof

By summing the equation in (2.1), integrating on $\Omega$ and using (A3) we have

$$\begin{aligned} \frac{d}{dt}\sum _{i=1}^{m}\int _{\Omega }c_iu_i^{\varepsilon } \,dx \le \int _{\Omega }\left( K_1 \sum _{i=1}^{m}u_i^{\varepsilon }(x,t) + K_2\right) \,dx. \end{aligned}$$

The classical Gronwall inequality gives the desired estimate. $\square$

The following $L^p$-energy function has been developed in [12, 23]. We write $\mathbb {Z}_{+}^m$ as the set of all *m* tuples of nonnegative integers. Addition and scalar multiplication by nonnegative integers of elements in $\mathbb {Z}_{+}^m$ is understood in the usual manner. If $\beta =\left( \beta _1, \ldots , \beta _m\right) \in \mathbb {Z}_{+}^m$ and $p \in \mathbb {Z}_+$, then we define $\beta ^p=\left( \left( \beta _1\right) ^p, \ldots ,\left( \beta _m\right) ^p\right)$. Also, if $\alpha =\left( \alpha _1, \ldots , \alpha _m\right) \in$ $\mathbb {Z}_{+}^m$, then we define $|\alpha |=\sum _{i=1}^m \alpha _i$. Finally, if $z=\left( z_1, \ldots , z_m\right) \in \mathbb {R}_{+}^m$ and $\alpha =$ $\left( \alpha _1, \ldots , \alpha _m\right) \in \mathbb {Z}_{+}^m$, then we define $z^\alpha =z_1^{\alpha _1} \cdots z_m^{\alpha _m}$, where we interpret $0^0$ to be 1. For any $2\le p \in {\mathbb {N}}$, we build our $L^p$-energy function of the form

2.2 $$\begin{aligned} \mathscr {L}_p[u](t)=\int _{\Omega } \mathscr {H}_p[u](t) \,d x, \end{aligned}$$

where

2.3 $$\begin{aligned} \mathscr {H}_p[u](t)=\sum _{\beta \in \mathbb {Z}_{+}^m,|\beta |=p}\begin{pmatrix}p \\ \beta \end{pmatrix} \theta ^{\beta ^2} u(t)^\beta \end{aligned}$$

with

$$\begin{aligned} \begin{pmatrix}p \\ \beta \end{pmatrix}=\frac{p !}{\beta _{1} ! \cdots \beta _{m} !} \end{aligned}$$

and $\theta =\left( \theta _1, \ldots , \theta _m\right)$, where $\theta _1, \ldots , \theta _m$ are positive real numbers which will be determined later. For $p=0,1,2$, one can write these functions explicitly as

$$\begin{aligned} \mathscr {H}_0[u](t)=1 \text{ and } \mathscr {H}_1[u](t)=\sum _{j=1}^m \theta _j u_j(t) \end{aligned}$$

and

$$\begin{aligned} \mathscr {H}_2[u](t)=\sum _{i=1}^m \theta _i^4 u_i(t)^2+2 \sum _{i=1}^{m-1} \sum _{j=i+1}^m \theta _i \theta _j u_i(t) u_j(t). \end{aligned}$$

Thanks to the nonnegativity of the solution, we have

$$\begin{aligned} \mathscr {L}_p[u](t) \sim \sum _{i=1}^m\Vert u_i(t)\Vert _{L^p(\Omega )}^p ~\text { for } p\ge 1. \end{aligned}$$

This will allow us to use $\mathscr {L}_p[u](t)$ to obtain a priori estimates on *u* for each $2 \le p \in \mathbb {N}$. We will need two technical lemmas.

#### Lemma 2.4

([12], Lemma 4.1) Suppose $m \in \mathbb {N}, \theta =\left( \theta _1, \ldots , \theta _m\right)$, where $\theta _1, \ldots , \theta _m$ are positive real numbers, $\beta \in \mathbb {Z}_{+}^m$, and $\mathscr {H}_p[u]$ is defined in (2.3). Then

$$\begin{aligned} \frac{\partial }{\partial t} \mathscr {H}_0[u](t)=0, \quad \frac{\partial }{\partial t} \mathscr {H}_1[u](t)=\sum _{j=1}^m \theta _j \frac{\partial }{\partial t} u_j(t) \end{aligned}$$

and for $p \in \mathbb {N}$ such that $p \ge 2$,

$$\begin{aligned} \frac{\partial }{\partial t} \mathscr {H}_p[u](t)=\sum _{|\beta |=p-1}\begin{pmatrix}p \\ \beta \end{pmatrix} \theta ^{\beta ^2} u(t)^\beta \sum _{j=1}^m \theta _j^{2 \beta _j+1} \frac{\partial }{\partial t} u_j(t). \end{aligned}$$

#### Lemma 2.5

([12], Lemma 4.2) Suppose $m \in \mathbb {N}, \theta =\left( \theta _1, \ldots , \theta _m\right)$, where $\theta _1, \ldots , \theta _m$ are positive real numbers, and let $\mathscr {H}_p[u]$ be defined in (2.3). If $p \in \mathbb {N}$ such that $p \ge 2$, then

$$\begin{aligned} \begin{aligned}&\sum _{|\beta |=p-1}\begin{pmatrix}p \\ \beta \end{pmatrix} \theta ^{\beta ^2} \sum _{k=1}^m \theta _k^{2 \beta _k+1}\left( A_k \nabla u_k\right) \cdot \nabla u^\beta \\ {}&\quad =\sum _{|\beta |=p-2}\begin{pmatrix}p \\ {} &{} \beta \end{pmatrix} \theta ^{\beta ^2} u^{\beta } \sum _{k=1}^m \sum _{l=1}^m C_{k, l}(\beta )\left( A_k \nabla u_k\right) \cdot \nabla u_l, \end{aligned} \end{aligned}$$

where

$$\begin{aligned} C_{k, l}(\beta )=\left\{ \begin{array}{cc} \theta _k^{2 \beta _k+1} \theta _l^{2 \beta _l+1}, &{} k \ne {l}, \\ \theta _k^{4 \beta _k+4}, &{} k= {l}. \end{array}\right. \end{aligned}$$

Next, we need the following functional inequality.

#### Lemma 2.6

Suppose $\Omega \subset \mathbb {R}^N$ such that the Gagliardo-Nirenberg inequality is satisfied and basic trace theorems apply (for instance $\Omega$ has a Lipschitz boundary). Let $a\ge 1$, $p\ge 2a$, $w: \overline{\Omega }\rightarrow \mathbb {R}_+$ such that $w^{\frac{b+p}{2}}\in W^{1,2}(\Omega )$ and there exists $K\ge 0$ such that $\Vert w\Vert _{L^a(\Omega )}\le K$. If $1\le r<1+b+ \frac{2a}{N}$ and $b\ge 0$, then there exists $C_{\varepsilon }\ge 0$ (depending on $p,\varepsilon , r, a, b, K, \Omega ,$ but independent of *w*) such that

$$\begin{aligned} \int _{\Omega }w^{p-1+r} \,dx +\int _{\Omega }w^{b+p}\,dx \le \varepsilon \left( \int _{\Omega } w^{p-2+b}|\nabla w|^{2}\,dx+\int _{\Omega } w^{b+p}\,dx\right) +C_{\varepsilon }. \end{aligned}$$

#### Proof

By using Sobolev’s embedding we have

2.4 $$\begin{aligned} \begin{aligned} \int _{\Omega }&w^{p-2+b}|\nabla w|^{2}\,dx+\int _{\Omega } w^{b+p}\,dx\\ {}&=\left( \frac{2}{b+p}\right) ^{2}\int _{\Omega } |\nabla w^{\frac{b+p}{2}}|^{2}\,dx+\int _{\Omega } (w^{\frac{b+p}{2}})^{2}\,dx\\ {}&\ge \min \left\{ \left( \frac{2}{b+p}\right) ^{2}, 1\right\} \Vert w^{\frac{b+p}{2}}\Vert ^{2}_{H^{1}(\Omega )}. \end{aligned} \end{aligned}$$

Thus we have

2.5 $$\begin{aligned} \int _{\Omega }w^{b+p}\,dx\le \varepsilon \left( \int _{\Omega } w^{p-2+b}|\nabla w|^{2}\,dx+ \int _{\Omega } w^{b+p}\,dx\right) +C(p,\varepsilon ,b,K). \end{aligned}$$

Since

$$\begin{aligned} \int _{\Omega }w^{p-1+r}\,dx=\Vert w^{\frac{p-1+r}{2}}\Vert ^2_{L^{2}(\Omega )}, \end{aligned}$$

if $r<b+1$. Then we have

$$\begin{aligned} \int _{\Omega }w^{p-1+r} \,dx \le \varepsilon \int _{\Omega } w^{b+p}\,dx +C_{\varepsilon }. \end{aligned}$$

Next we consider $r\ge b+1$. Thanks to the Gagliardo-Nirenberg inequality, we have

2.6 $$\begin{aligned} \begin{aligned} \int _{\Omega }w^{p-1+r}\,dx&=\Vert w^{\frac{b+p}{2}}\Vert _{L^{\frac{2(r+p-1)}{b+p}} (\Omega )}^{\frac{2(r+p-1)}{b+p}}\\ {}&\le C \Vert \nabla (w^{\frac{b+p}{2}})\Vert _{L^{2}(\Omega )}^{\frac{2(r+p-1)}{b+p}\alpha } \Vert w^{\frac{b+p}{2}}\Vert _{L^{1} (\Omega )}^{\frac{2(r+p-1)}{b+p}(1-\alpha )}, \end{aligned} \end{aligned}$$

where

$$\begin{aligned} \begin{aligned} \frac{b+p}{2(r+p-1)}=\alpha (\frac{1}{2}-\frac{1}{N})+1-\alpha . \end{aligned} \end{aligned}$$

It follows that

$$\begin{aligned} \begin{aligned} \alpha =\frac{N[2(r+p-1)-(b+p)]}{(r+p-1)(N+2)} \end{aligned} \end{aligned}$$

and

$$\begin{aligned} \begin{aligned} 1-\alpha =\frac{(r+p-1)(2-N)+N(b+p)}{(r+p-1)(N+2)}. \end{aligned} \end{aligned}$$

Since $1\le r<1+b+\frac{2a}{N}$, we find

$$\begin{aligned} \alpha \frac{2(r+p-1)}{b+p}<2. \end{aligned}$$

We can use Young’s inequality to estimate

2.7 $$\begin{aligned} \begin{aligned} \Vert w^{\frac{b+p}{2}}\Vert _{L^{\frac{2(r+p-1)}{b+p}}(\Omega )}^{\frac{2(r+p-1)}{b+p}}&\le \varepsilon \Vert \nabla \left( w^{\frac{b+p}{2}}\right) \Vert _{L^{2}(\Omega )}^{2}+C_{\varepsilon }\Vert w^{\frac{b+p}{2}}\Vert _{L^{1} (\Omega )}^{\frac{(1-\alpha )2(r+p-1)}{b+p-(r+p-1)\alpha }}, \end{aligned} \end{aligned}$$

where

2.8 $$\begin{aligned} \begin{aligned} C_{\varepsilon }\Vert w^{\frac{b+p}{2}}\Vert _{L^{1} (\Omega )}^{\frac{(1-\alpha )2(r+p-1)}{b+p-(r+p-1)\alpha }}=C_{\varepsilon }\Vert w \Vert _{L^{\frac{b+p}{2}} (\Omega )}^{\frac{(1-\alpha )(b+p)(r+p-1)}{b+p-(r+p-1)\alpha }}. \end{aligned} \end{aligned}$$

If $b+p=2a$, then this term is bounded by a constant depending on *K*, since $\Vert w\Vert _{L^a(\Omega )}\le K$. If $b+p>2a$, we use an interpolation inequality to have

2.9 $$\begin{aligned} \begin{aligned} C_{\varepsilon }\Vert w\Vert _{L^{\frac{b+p}{2}} (\Omega )}\le C_{\varepsilon }\Vert w\Vert ^{\theta }_{L^{p+r-1} (\Omega )}\Vert w\Vert ^{1-\theta }_{L^{a} (\Omega )}\le C_{\varepsilon , K} \Vert w\Vert ^{\theta }_{L^{p+r-1} (\Omega )}, \end{aligned} \end{aligned}$$

where $$\theta \in (0,1)$$ satisfies

$$\begin{aligned} \frac{2}{b+p}=\frac{\theta }{p+r-1}+\frac{1-\theta }{a}. \end{aligned}$$

Note that

2.10 $$\begin{aligned} \theta \frac{(1-\alpha )(b+p)(r+p-1)}{b+p-(r+p-1)\alpha }<p+r-1 \end{aligned}$$

due to $r<b+1+ \frac{2a}{N}$ and $b\le r-1<2r$.

From (2.8)–(2.10) and Young’s inequality, it follows that

2.11 $$\begin{aligned} \begin{aligned} C_{\varepsilon }\Vert w \Vert ^{\frac{(1-\alpha )(b+p)(r+p-1)}{b+p-(r+p-1)\alpha }}_{L^{\frac{b+p}{2}} (\Omega )}&\le C_{\varepsilon , K} \Vert w \Vert ^{\frac{(1-\alpha )(b+p)(r+p-1)}{b+p-(r+p-1)\alpha }\theta }_{L^{p+r-1} (\Omega )}\\&\le \frac{1}{2} \Vert w \Vert ^{p+r-1}_{L^{p+r-1}(\Omega )}+C(p,\varepsilon ,b,K). \end{aligned} \end{aligned}$$

Inserting this into (2.6), we get

$$\begin{aligned} \begin{aligned} \Vert w\Vert ^{p+r-1}_{L^{p+r-1}(\Omega )}\le 2\varepsilon \Vert w^{\frac{b+p}{2}}\Vert ^{2}_{W^{1,2}(\Omega )} +C(p,\varepsilon ,b,K). \end{aligned} \end{aligned}$$

Combine this with (2.4) and (2.5) leads to the desired estimate

$$\begin{aligned}&\int _{\Omega }w^{p-1+r} +\int _{\Omega }w^{b+p}\,dx\\ {}&\qquad \le \varepsilon \left( \int _{\Omega } w^{p-2+b}|\nabla w|^{2}\,dx+\int _{\Omega } w^{b+p}\,dx\right) +C(p,\varepsilon ,b,K). \end{aligned}$$

$$\square $$

The following lemma shows results for the intermediate sum condition (A4), which is crucial for constructing the $L^p$ energy function.

#### Lemma 2.7

([12], Lemma 2.4) Assume (A4). Then there exist componentwise increasing functions $g_i: \mathbb {R}^{m-i} \rightarrow \mathbb {R}_{+}$ for $i=1, \ldots , m-1$ such that if $\theta =\left( \theta _1, \ldots , \theta _m\right) \in (0, \infty )^m$ satisfies $\theta _m>0$ and $\theta _i \ge g_i\left( \theta _{i+1}, \ldots , \theta _m\right)$ for all $i=1, \ldots , m-1$, then

$$\begin{aligned} \sum _{i=1}^m \theta _i f_i^{\varepsilon }\left( x, t, u^{\varepsilon }\right) \le K_\theta \left( 1+\sum _{i=1}^m\left( u_i^{\varepsilon }\right) ^r\right) \quad \forall \left( x, t, u^{\varepsilon }\right) \in \Omega \times \mathbb {R}_{+} \times \mathbb {R}_{+}^m \end{aligned}$$

for some constant $K_\theta$ depending on $\theta , g_i$, and $K_3$ in (A4).

We now use the $L^p$-energy functions to obtain the $L^p$-estimates of $u^{\varepsilon }$.

#### Lemma 2.8

Assume (1.3), (1.4), (Q1), (Q2), (A1), (A2), (A3) and (A4) with $1\le r<1+b+\frac{2}{N}$. Then for any $1 \le p<\infty$ and any $T>0$, there exists a constant $C_{T, p}$ depending on *T*, *p* and other parameters such that

$$\begin{aligned} \sup _{t \in (0, T)}\Vert u_i^{\varepsilon }(t)\Vert _{L^p(\Omega )} \le C_{T, p} \quad \forall i=1, \ldots , m. \end{aligned}$$

#### Proof

Let $u^{\varepsilon }$ solve (2.1), and $\mathscr {L}_p(t):=\mathscr {L}_p\left[ u^{\varepsilon }\right] (t)$ be defined in (2.2). Then

$$\begin{aligned} \begin{aligned} \mathscr {L}_p^{\prime }(t)=&\int _{\Omega } \sum _{|\beta |=p-1}\begin{pmatrix}p \\ \beta \end{pmatrix} \theta ^{\beta ^2} u^{\varepsilon }(x, t)^\beta \sum _{k=1}^m \theta _k^{2 \beta _k+1} \frac{\partial }{\partial t} u_k^{\varepsilon }(x, t)\, d x \\ =&\int _{\Omega } \sum _{|\beta |=p-1}\begin{pmatrix}p \\ \beta \end{pmatrix} \theta ^{\beta ^2} u^{\varepsilon }(x, t)^\beta \sum _{k=1}^m \theta _k^{2 \beta _k+1} \\ {}&\times \left[ \nabla \cdot \left( D_k(x, t)\,\Phi (u^{\varepsilon })\, \nabla u_k^{\varepsilon }(x, t)\right) +f^{\varepsilon }_k\left( u^{\varepsilon }(x, t)\right) \right] \, d x\\ =&\int _{\Omega } \sum _{|\beta |=p-1} \begin{pmatrix}p \\ \beta \end{pmatrix} \theta ^{\beta ^2} u^{\varepsilon }(x, t)^\beta \sum _{k=1}^m \theta _k^{2 \beta _k+1}\nabla \cdot \left( D_k(x, t) \,\Phi (u^{\varepsilon })\, \nabla u_k^{\varepsilon }(x, t)\right) \, d x\\ {}&+ \int _{\Omega } \sum _{|\beta |=p-1}\begin{pmatrix}p \\ \beta \end{pmatrix} \theta ^{\beta ^2} u^{\varepsilon }(x, t)^\beta \sum _{k=1}^m \theta _k^{2 \beta _k+1} f^{\varepsilon }_k\left( u^{\varepsilon }(x, t)\right) \, d x\\ =:&(I)+(II). \end{aligned} \end{aligned}$$

For (I), we apply Lemma 2.5 and integration by parts, we have

$$\begin{aligned} \begin{aligned}&(I):= \int _{\Omega } \sum _{|\beta |=p-1} \begin{pmatrix}p \\ \beta \end{pmatrix} \theta ^{\beta ^2} u^{\varepsilon }(x, t)^\beta \sum _{k=1}^m \theta _k^{2 \beta _k+1}\nabla \cdot \left( D_k(x, t) \,\Phi (u^{\varepsilon })\, \nabla u_k^{\varepsilon }(x, t)\right) \, d x\\ {}&\quad \;=-\int _{\Omega } \sum _{|\beta |=p-2} \begin{pmatrix}p \\ \beta \end{pmatrix} \theta ^{\beta ^2} u^{\varepsilon }(x, t)^\beta \,\Phi (u^{\varepsilon })\, \sum _{k=1}^m \sum _{l=1}^m C_{k, l}(\beta )\left( D_k(x,t) \nabla u_k^{\varepsilon }\right) \cdot \nabla u_l^{\varepsilon } \,dx \end{aligned} \end{aligned}$$

with

$$\begin{aligned} C_{k, l}(\beta )=\left\{ \begin{array}{cc} \theta _k^{2 \beta _k+1} \theta _l^{2 \beta _l+1}, &{} k \ne l, \\ \theta _k^{4 \beta _{k}+4}, &{} k=l. \end{array}\right. \end{aligned}$$

For a given $\beta$ with $|\beta |=p-2$, create an $m N \times m N$ matrix $B(\beta )$ made up of $m^2$ blocks $B_{k, l}(\beta )$, each of size $N \times N$, where

$$\begin{aligned} B_{k, l}(\beta )=\frac{1}{2} C_{k, l}(\beta )\left( D_k+D_l\right) . \end{aligned}$$

Note that for each $k=1, \ldots , m$,

$$\begin{aligned} B_{k, k}(\beta )=\theta _k^{4 \beta _k+4} D_k. \end{aligned}$$

Also,

$$\begin{aligned} I=-\int _{\Omega } \sum _{|\beta |=p-2}\begin{pmatrix}p \\ \beta \end{pmatrix} \theta ^{\beta ^2} u^{\varepsilon }(x, t)^{\beta } \,\Phi (u^{\varepsilon })\, \nabla u^{\varepsilon }(x, t)^T B(\beta ) \nabla u^{\varepsilon }(x, t)\, dx, \end{aligned}$$

where $\nabla u^{\varepsilon }(x, t)$ is a column vector of size $m N \times 1$, and for $j=1, \ldots , m$, entries $N(j-1)+1$ to *Nj* of $\nabla u^{\varepsilon }(x, t)$ are $\nabla u_j^{\varepsilon }(x, t)$. We claim that if all of the entries in $\theta$ are sufficiently large, then $B(\beta )$ is positive definite. In fact, it is a simple matter to show it is positive definite if and only if the $m N \times m N$ matrix ${\widetilde{B}}(\beta )$ made up of $N \times N$ blocks

$$\begin{aligned} {\widetilde{B}}_{k, l}(\beta )=\left\{ \begin{array}{cc} \theta _k^2 D_k, &{} k=l \\ \frac{1}{2}\left( D_k+D_l\right) , &{} k \ne l \end{array}\right. \end{aligned}$$

is positive definite. However, if we recall the uniform positive definiteness of the matrices $D_k$, we can show that if $\theta _i$ is sufficiently large for each *i*, then we have what we need. Consequently, returning to the above, we can show there exists $\alpha _p>0$ so that

2.12 $$\begin{aligned} \begin{aligned} \mathscr {L}_p^{\prime }(t)+&\alpha _p \sum _{k=1}^m \int _{\Omega }u_k^{\varepsilon }(x,t)^{b+p-2} \left| \nabla u^{\varepsilon }_k(x, t)\right| ^2 \,dx \\ {}&\quad \le \int _{\Omega } \sum _{|\beta |=p-1} \begin{pmatrix}p \\ \beta \end{pmatrix} \theta ^{\beta ^2} u^{\varepsilon }(x, t)^\beta \sum _{k=1}^m \theta _k^{2 \beta _k+1}f_k^{\varepsilon }\left( u^{\varepsilon }(x, t)\right) \, dx, \end{aligned} \end{aligned}$$

where we used (Q2).

From (A4) and Lemma 2.7, we choose the components of $\theta = (\theta _i)$ inductively so that $\theta _i$ are sufficiently large that the previous positive definiteness condition of ${\widetilde{B}}_{k, l}(\beta )$ is satisfied, and

2.13 $$\begin{aligned} \begin{aligned} \theta _i\ge g_i(\theta _{i+1}^{2p-1},\ldots ,\theta _{m}^{2p-1}) \quad \text {for}\quad i=1,\ldots ,m-1, \end{aligned} \end{aligned}$$

where $g_i$ are functions constructed in Lemma 2.7. Note that $\theta _i \le \theta _i^{2\beta _i + 1} \le \theta _i^{2p - 1}$. Since $g_i$ is componentwise increasing, the relation (2.13) implies

$$\begin{aligned} \theta _i^{2\beta _i + 1} \ge g_i\left( \theta _{i+1}^{2\beta _{i+1}+1}, \ldots , \theta _m^{2\beta _m + 1}\right) , \quad \forall i=1,\ldots , m-1. \end{aligned}$$

Now we can apply Lemma 2.7, to obtain some $K_{\widetilde{\theta }}$ so that for all $\beta \in \mathbb {Z}_+$ with $|\beta |=p-1$, we have

$$\begin{aligned} \begin{aligned} \sum _{i=1}^{m}\theta _{i}^{2\beta _i+1}f^{\varepsilon }_i(x,t,u^{\varepsilon })\le K_{\widetilde{\theta }}\left( 1+\sum _{i=1}^{m}(u^{\varepsilon }_i)^r\right) , \quad \forall (x,t,u)\in \Omega \times \mathbb {R}_+\times \mathbb {R}^m_+. \end{aligned} \end{aligned}$$

It follows that there exists $C_p>0$, such that (2.12) implies

$$\begin{aligned} \begin{aligned} \mathscr {L}_p^{\prime }(t)+&\alpha _p \sum _{k=1}^m \int _{\Omega }u_k^{\varepsilon }(x,t)^{b+p-2} \left| \nabla u^{\varepsilon }_k(x, t)\right| ^2 \,dx \\ {}&\quad \le C\left( \int _{\Omega }\sum _{i=1}^{m}u_i^{\varepsilon }(x,t)^{p-1+r}\,dx+1\right) . \end{aligned} \end{aligned}$$

Thus, we have

2.14 $$\begin{aligned} \begin{aligned} \mathscr {L}_p^{\prime }(t)+&\alpha _p \sum _{k=1}^m \int _{\Omega }\left( u_k^{\varepsilon }(x,t)^{b+p-2} \left| \nabla u^{\varepsilon }_k(x, t)\right| ^2 +u_i^{\varepsilon }(x,t)^{b+p}\right) \,dx\\ {}&\quad \le C\left( \int _{\Omega }\sum _{i=1}^{m}\left( u_i^{\varepsilon }(x,t)^{p-1+r} + u_i^{\varepsilon }(x,t)^{b+p}\right) \,dx+1\right) . \end{aligned} \end{aligned}$$

Combining Lemma 2.6, we get

2.15 $$\begin{aligned} \begin{aligned} \mathscr {L}_p^{\prime }(t)+&\frac{\alpha _p}{2} \sum _{k=1}^m \int _{\Omega }\left( u_k^{\varepsilon }(x,t)^{b+p-2} \left| \nabla u^{\varepsilon }_k(x, t)\right| ^2 +u_i^{\varepsilon }(x,t)^{b+p}\right) \,dx \le C(T,p). \end{aligned}\nonumber \\ \end{aligned}$$

This implies

$$\begin{aligned} \begin{aligned} \mathscr {L}_p^{\prime }(t) + \sigma \mathscr {L}_p(t) \le C(T,p). \end{aligned} \end{aligned}$$

Thus, we have

$$\begin{aligned} \sup _{t \in (0, T)}\Vert u_i^{\varepsilon }(t)\Vert _{L^p(\Omega )} \le C_{T, p} \quad \forall i=1, \ldots , m. \end{aligned}$$

This finishes the proof of Lemma 2.8. $\square$

#### Lemma 2.9

Assume (1.3), (1.4), (Q1), (Q2), (A1), (A2), (A3), (A5) and (A4) with $1\le r<1+b+\frac{2}{N}$. Then for any $T>0$, the solution of (2.1) is bounded in $L^{\infty }$ in time, i.e.,

$$\begin{aligned} \Vert u_i^{\varepsilon }(t)\Vert _{L^{\infty }(Q_T)} \le C_{T} \quad \forall i=1, \ldots , m \end{aligned}$$

for some constant $C_T$ depending on *T* and independent of $\varepsilon >0$.

#### Proof

The proof is similar to the proof of Lemma 2.3 in Ref. [9]. For the convenience of reading, we give the specific proof process in the Appendix A. $\square$

### Passing to the limit—Global existence

#### Lemma 2.10

Assume (1.3). For any $k>0$, we have

$$\begin{aligned} \int ^T_0\int _{\Omega }\chi _{\{|u^{\varepsilon }_i|\le k\}} \Phi (u^{\varepsilon }) |\nabla u^{\varepsilon }_i|^2 \,dxdt \le \frac{k}{\lambda } \left( \Vert u_0\Vert _{L^{1}(\Omega )}+\Vert f^{\varepsilon }_i(u^{\varepsilon })\Vert _{L^{1}(Q_T)}\right) . \end{aligned}$$

#### Proof

Let $T_k(z)$ be defined

$$\begin{aligned} T_k(z)={\left\{ \begin{array}{ll} -k &{} if z\le -k,\\ z &{} if -k<z<k,\\ k &{} if z\ge k. \end{array}\right. } \end{aligned}$$

Define $S_k(z)=\int ^z_0 T_k(\tau ) \,d\tau$ and multiply (2.1) by $T_k(u^{\varepsilon }_i)$ we obtain

$$\begin{aligned} \begin{aligned}&\int _{\Omega }S_k(u_i^{\varepsilon })\,dx + \int ^T_0\int _{\Omega }D_i(x,t) \Phi (u^{\varepsilon }) \nabla u_i^{\varepsilon } \cdot \nabla (T_k(u^{\varepsilon }_i))\, dxdt\\ {}&\quad =\int _{\Omega }S_k(u_0^{\varepsilon })\,dx + \int ^T_0\int _{\Omega }f^{\varepsilon }_i(u^{\varepsilon })T_k(u^{\varepsilon }_i)\,dxdt. \end{aligned} \end{aligned}$$

By applying the properties of $T_k$ and $S_k$, the right hand is bounded by

$$\begin{aligned} \begin{aligned} \int _{\Omega }S_k(u_0^{\varepsilon })\,dx + \int ^T_0\int _{\Omega }f^{\varepsilon }_i(u^{\varepsilon })T_k(u^{\varepsilon }_i)\,dxdt\le k \left( \Vert u_0\Vert _{L^{1}(\Omega )}+\Vert f_i^{\varepsilon }\Vert _{L^{1}(Q_T)} \right) . \end{aligned} \end{aligned}$$

From (1.3) and $\nabla (T_k(u^{\varepsilon }_i))=\chi _{\{|u^{\varepsilon }_i|\le k\}} \nabla u^{\varepsilon }_i$, it follows that

$$\begin{aligned} \begin{aligned} \int ^T_0\int _{\Omega }D_i(x,t) \Phi (u^{\varepsilon }) \nabla u_i^{\varepsilon } \cdot \nabla (T_k(u^{\varepsilon }_i))\, dxdt&\ge \lambda \int ^T_0\int _{\Omega }\chi _{\{|u^{\varepsilon }_i|\le k\}} \Phi (u^{\varepsilon }) |\nabla u_i^{\varepsilon }|^2 \, dxdt. \end{aligned} \end{aligned}$$

Finally, by using $S_k(u^{\varepsilon }_i)\ge 0$ we obtain our desired estimate. $\square$

#### Lemma 2.11

Assume (1.3). For any $\beta >0$, there exists constants *C* such that

2.16 $$\begin{aligned} \int ^T_0\int _{\Omega }\frac{\Phi (u^{\varepsilon }) |\nabla u^{\varepsilon }_i|^2}{(1+|u^{\varepsilon }_i|)^{1+\beta } } \,dxdt \le C \left( \Vert u_0\Vert _{L^{1}(\Omega )} + \Vert f^{\varepsilon }_i(u^{\varepsilon })\Vert _{L^{1}(Q_T)}\right) . \end{aligned}$$

#### Proof

Let $M:=\Vert u_0\Vert _{L^{1}(\Omega )}+\Vert f^{\varepsilon }_i(u^{\varepsilon })\Vert _{L^{1}(Q_T)}$, we can apply Lemma 2.10 to obtain

$$\begin{aligned} \begin{aligned} \int ^T_0\int _{\Omega } \frac{ \Phi (u^{\varepsilon }) |\nabla u^{\varepsilon }_i|^2}{(1+|u^{\varepsilon }_i|)^{1+\beta } } \,dxdt&=\sum ^{\infty }_{j=0} \int ^T_0\int _{\Omega }\textbf{1}_{\{2^j-1\le |u^{\varepsilon }_i|\le 2^{j+1}-1\}} \frac{ \Phi (u^{\varepsilon }) |\nabla u^{\varepsilon }_i|^2}{(1+|u^{\varepsilon }_i|)^{1+\beta } } \,dxdt\\ {}&\le \sum ^{\infty }_{j=0}2^{-j(1+\beta )} \int ^T_0\int _{\Omega }\textbf{1}_{\{|u^{\varepsilon }_i|\le 2^{j+1}-1\}} \Phi (u^{\varepsilon }) |\nabla u^{\varepsilon }_i|^2 \,dxdt\\ {}&\le \frac{M}{\lambda } \sum ^{\infty }_{j=0}2^{-j(1+\beta )} 2^{j+1} \le \frac{2M}{\lambda } \sum ^{\infty }_{j=0}(2^{-\beta })^{j}. \end{aligned} \end{aligned}$$

Thus (2.16) holds for $C:=\frac{2}{\lambda } \sum ^{\infty }_{j=0}(2^{-\beta })^{j}$, which is finite since $\beta >0$. $\square$

#### Proof of Theorem 1.1-Global existence

From Sect. 2.2 we have the following bound

$$\begin{aligned} \Vert u_i^{\varepsilon }(t)\Vert _{L^{\infty }(Q_T)} \le C_{T} \quad \forall i=1, \ldots , m. \end{aligned}$$

Due to the polynomial growth (A5), for any $i=1, \ldots , m$, we can get

2.17 $$\begin{aligned} {\Vert f_i(u_i^{\varepsilon }(t))\Vert _{L^{\infty }(Q_T)}\le K_4\Vert ( 1+\sum _{i=1}^{m}(u^{\varepsilon }_i)^l )\Vert _{L^{\infty }(Q_T)} \le C_{T}.} \end{aligned}$$

By multiply (2.1) by $u_i^{\varepsilon }$ then integrating on $Q_T$ gives

$$\begin{aligned} \begin{aligned} \frac{1}{2}\Vert u^{\varepsilon }_i(T)\Vert ^2_{L^{2}(\Omega )}&+\int ^T_0\int _{\Omega }D_i(x,t) \Phi (u^{\varepsilon }) \nabla u_i^{\varepsilon } \cdot \nabla u_i^{\varepsilon } \,dxdt\\&=\frac{1}{2}\Vert u^{\varepsilon }_i(0)\Vert ^2_{L^{2}(\Omega )}+\int ^T_0\int _{\Omega }f_i^{\varepsilon }(u^{\varepsilon }) u_i^{\varepsilon } \,dxdt\\ {}&\le \frac{1}{2}\Vert u^{\varepsilon }_i(0)\Vert ^2_{L^{2}(\Omega )}+\Vert f_i^{\varepsilon }(u^{\varepsilon }) \Vert _{L^{\infty }(Q_T)}\Vert u_i^{\varepsilon }\Vert _{L^{1}(Q_T)}\\ {}&\le C\left( T, |\Omega |, \Vert u_{i,0}\Vert _{L^{\infty }(\Omega )}\right) . \end{aligned} \end{aligned}$$

By (1.3), (Q2) and Hölder inequality give

$$\begin{aligned} \begin{aligned} \int ^T_0&\int _{\Omega }D_i(x,t) \Phi (u^{\varepsilon }) \nabla u_i^{\varepsilon } \cdot \nabla u_i^{\varepsilon } \,dxdt \\ {}&\ge \lambda M_i \int ^T_0\int _{\Omega }(u_i^{\varepsilon })^b |\nabla u_i^{\varepsilon }|^2\,dxdt \\ {}&=\lambda M_i (\frac{2}{b+2})^2 \int ^T_0\int _{\Omega }|\nabla ((u_i^{\varepsilon })^{\frac{b+2}{2}})|^2\,dxdt\\ {}&=\lambda M_i (\frac{2}{b+2})^2 \Vert \nabla (( u_i^{\varepsilon })^{\frac{b+2}{2}})\Vert _{L^{2}(Q_T)}^2. \end{aligned} \end{aligned}$$

In particular

2.18 $$\begin{aligned} \begin{aligned} \Vert \nabla ((u_i^{\varepsilon })^{\frac{b+2}{2}})\Vert _{L^{2}(Q_T)}\le C\left( T, |\Omega |, \Vert u_{i,0}\Vert _{L^{\infty }(\Omega )}\right) . \end{aligned} \end{aligned}$$

By testing the equation (2.1) with $\varphi \in L^2(0,T; H^1(\Omega ))$, we have

$$\begin{aligned} \begin{aligned}&\int ^T_0\int _{\Omega }\partial _tu_i^{\varepsilon } \varphi =-\int ^T_0\int _{\Omega }D_i(x,t) \Phi (u^{\varepsilon }) \nabla u_i^{\varepsilon } \cdot \nabla \varphi +\int ^T_0\int _{\Omega }f_i^{\varepsilon }(u^{\varepsilon }) \varphi \\ {}&\quad \quad \quad \quad \quad \;\;=-\int ^T_0\int _{\Omega }D_i(x,t) \Phi (u^{\varepsilon })^{\frac{1}{2}} (1+u_i^{\varepsilon })^{\frac{1+\beta }{2}} \frac{ \Phi (u^{\varepsilon })^{\frac{1}{2}}\nabla u_i^{\varepsilon }}{(1+u_i^{\varepsilon })^{\frac{1+\beta }{2}}} \cdot \nabla \varphi +\int ^T_0\int _{\Omega }f_i^{\varepsilon }(u^{\varepsilon }) \varphi \\ {}&\quad \quad \quad \quad \quad \;\;\le \Vert D_i(x,t)\Vert _{L^{\infty }(Q_T)} \Vert \Phi (u^{\varepsilon })\Vert ^{\frac{1}{2}}_{L^{\infty }(Q_T)} (1+\Vert u_i^{\varepsilon }\Vert _{L^{\infty }(Q_T)})^{\frac{1+\beta }{2}}\\ {}&\qquad \quad \quad \quad \quad \quad \times \Vert \frac{\Phi (u^{\varepsilon })^{\frac{1}{2}}|\nabla u_i^{\varepsilon }|}{(1+u_i^{\varepsilon })^{\frac{1+\beta }{2}}}\Vert _{L^{2}(Q_T)} \Vert \nabla \varphi \Vert _{L^{2}(Q_T)}\\ {}&\qquad \quad \quad \quad \quad \quad +\Vert f_i^{\varepsilon }(u^{\varepsilon })\Vert _{L^{\infty }(Q_T)} (|\Omega | T)^{\frac{1}{2}}\Vert \varphi \Vert _{L^{2}(Q_T)}\\ {}&\quad \quad \quad \quad \quad \;\;\le C\left( T, |\Omega |,\Vert u_{i,0}\Vert _{L^{\infty }(\Omega )}, \Vert D_i(x,t)\Vert _{L^{\infty }(Q_T)}\right) \Vert \varphi \Vert _{L^2(0,T; H^1(\Omega ))}, \end{aligned} \end{aligned}$$

where we use (1.4), (Q3), (2.16) and (2.17). Thus we can get

2.19 $$\begin{aligned} \Vert \partial _t u_i^{\varepsilon }\Vert _{L^2(0,T; (H^1(\Omega ))')}\le C\left( T, |\Omega |, \Vert u_{i,0}\Vert _{L^{\infty }(\Omega )}\right) . \end{aligned}$$

From Lemma 2.9, (2.18) and (2.19), we can apply a nonlinear version of the well known Aubin-Lions Lemma (see e.g. [21, Theorem 1.1]) to ensure

2.20 $$\begin{aligned} u_i^{\varepsilon }{\mathop {\longrightarrow }\limits ^{\varepsilon \rightarrow 0}}u_i \quad \text {strongly in } L^{2}(Q_T) \end{aligned}$$

Thanks to the $L^{\infty }$ bound of Lemma 2.9, this convergence in fact holds in $L^{p}(Q_T)$ for any $1\le p<\infty$.

Since the sequence $(u_i^\varepsilon )^{\frac{b+2}{2}}$ is uniformly bounded in $L^2(0,T; H^{1}(\Omega ))$, there exists a subsequence (still denoted by $(u_i^\varepsilon )$) and $v\in L^2(0,T; H^{1}(\Omega ))$ such that

$$\begin{aligned} (u_i^\varepsilon )^{\frac{b+2}{2}} \rightharpoonup v \quad \text {weekly in } L^2(0,T; H^{1}(\Omega )). \end{aligned}$$

Now, we are ready to identify the weak limit *v* by the following computation: for testing functions $\varphi \in C^{\infty }_0(Q_T)$ we have

$$\begin{aligned} \int _0^T\int _{\Omega }\nabla v \cdot \varphi \,dxdt&= \lim _{\varepsilon \rightarrow 0} \int _0^T\int _{\Omega }\nabla (u_i^\varepsilon )^{\frac{b+2}{2}} \cdot \varphi \,dxdt\\ {}&= - \lim _{\varepsilon \rightarrow 0} \int _0^T\int _{\Omega }(u_i^\varepsilon )^{\frac{b+2}{2}} \cdot \nabla \varphi \,dxdt\\ {}&= - \int _0^T\int _{\Omega }u_i^{\frac{b+2}{2}} \cdot \nabla \varphi \,dxdt. \end{aligned}$$

This implies that the function $u_i^{\frac{b+2}{2}}$ is weakly differentiable with weak derivative *v*, i.e., $u_i^{\frac{b+2}{2}} = v$. Thus

2.21 $$\begin{aligned} (u_i^\varepsilon )^{\frac{b+2}{2}} \rightharpoonup u_i^{\frac{b+2}{2}} \quad \text {weekly in } L^2(0,T; H^{1}(\Omega )). \end{aligned}$$

It remains to pass to the limit $\varepsilon \rightarrow 0$ in the weak formulation of (2.1) with $\eta \in L^2(0,T; H^1(\Omega ))$,

2.22 $$\begin{aligned} \begin{aligned} \int ^T_0\langle \partial _t u_i^{\varepsilon }, \eta \rangle _{(H^1(\Omega ))', H^1(\Omega )} \,dt&+\int ^T_0\int _{\Omega }D_i(x,t) \Phi (u^{\varepsilon }) \nabla u_i^{\varepsilon }\cdot \nabla \eta \,dxdt\\ {}&=\int ^T_0\int _{\Omega }f_i^{\varepsilon }(u^{\varepsilon }) \eta \,dxdt. \end{aligned} \end{aligned}$$

The convergence of the first term on the left hand side and the term on the right hand side of (2.22) is immediate.

The convergence

2.23 $$\begin{aligned} \begin{aligned} \int ^T_0\int _{\Omega }D_i(x,t) \Phi (u^{\varepsilon }) \nabla u_i^{\varepsilon }\cdot \nabla \eta {\mathop {\longrightarrow }\limits ^{\varepsilon \rightarrow 0}} \int ^T_0\int _{\Omega }D_i(x,t) \Phi (u) \nabla u_i\cdot \nabla \eta \end{aligned} \end{aligned}$$

follows from $\frac{\Phi (u^{\varepsilon })}{(u^{\varepsilon }_i)^{\frac{b}{2}}} {\mathop {\longrightarrow }\limits ^{\varepsilon \rightarrow 0}} \frac{\Phi (u)}{u_i^{\frac{b}{2}} }$ a.e. in $Q_T$, Lemma 2.9 and the equation (2.21). Passing to the limit in the weak formulation (2.22), we have

2.24 $$\begin{aligned} \begin{aligned} \int ^T_0 \int _{\Omega }\partial _t u_i \eta \,dxdt&+\int ^T_0\int _{\Omega }D_i(x,t) \Phi (u) \nabla u_i\cdot \nabla \eta \,dxdt=\int ^T_0\int _{\Omega }f_i(u) \eta \,dxdt. \end{aligned} \end{aligned}$$

We obtain that $u=\left( u_1,\ldots ,u_m\right)$ is a global weak solution to (1.2) and additionally

$$\begin{aligned} \Vert u_i\Vert _{L^{\infty }(Q_T)}\le C_T, \quad \forall i=1,\ldots ,m. \end{aligned}$$

$$\square $$

## Uniform-in-time boundness

### Lemma 3.1

Assume (A1), (A2) and (A3) with either $K_1<0$ or $K_1=K_2=0$. Then, there exists a constant M independent of time such that

3.1 $$\begin{aligned} \sup _{t\ge 0}\Vert u_i(t)\Vert _{L^{1}(\Omega )}\le M:=\Vert u_{i0}\Vert _{L^{1}(\Omega )}, \quad \forall i=1,\ldots ,m. \end{aligned}$$

### Proof

Similar to the proof of Lemma 2.3, we have

$$\begin{aligned} \frac{d}{dt}\sum _{i=1}^{m}\int _{\Omega }c_iu_i \,dx \le \int _{\Omega }\left( K_1 \sum _{i=1}^{m}u_i^{\varepsilon }(x,t) + K_2\right) \,dx. \end{aligned}$$

Integrating over (*s*, *t*) we have

$$\begin{aligned} \sum _{i=1}^{m}c_i\int _{\Omega }u_i(x,t) \,dx&\le \sum _{i=1}^{m}c_i\int _{\Omega }u_i(x,s) \,dx \\&\quad +\int ^t_s\int _{\Omega }K_1 \sum _{i=1}^{m}u_i(x,r) \,dx dr+ K_2 |\Omega |(t-s) \end{aligned}$$

for all $$t>s\ge 0$$.

If $K_1=K_2=0$, the bound (3.1) follows immediately.

If $K_1<0$, we get for some constant $\sigma >0$ and for all $t>s\ge 0$,

3.2 $$\begin{aligned} \begin{aligned} \sum _{i=1}^{m}c_i\int _{\Omega }u_i(x,t) \,dx&+ \sigma \int ^t_s \left( \sum _{i=1}^{m}c_i\int _{\Omega }u_i(x,r) \,dx\right) dr \\ {}&\le \sum _{i=1}^{m}c_i\int _{\Omega }u_i(x,s) \,dx+ K_2 |\Omega |(t-s). \end{aligned} \end{aligned}$$

Define

$$\begin{aligned} \psi (t)= \sum _{i=1}^{m}c_i\int _{\Omega }u_i(x,t) \,dx ~\text { and } ~\phi (s) = \int ^t_s \left( \sum _{i=1}^{m}c_i\int _{\Omega }u_i(x,r) \,dx\right) dr. \end{aligned}$$

It follows from (3.2) that

$$\begin{aligned} \phi ^{\prime }(s)=-\sum _{i=1}^m c_i \int _{\Omega } u_i(\cdot , s) d x \le -\psi (t)-\sigma \phi (s)+K_2|\Omega |(t-s), \end{aligned}$$

which leads to

$$\begin{aligned} \left( e^{\sigma s} \phi (s)\right) ^{\prime }+e^{\sigma s} \psi (t) \le K_2|\Omega | e^{\sigma s}(t-s). \end{aligned}$$

Integrating with respect to *s* on (0, *t*), and using $\phi (t)=0$, we have

$$\begin{aligned} -\phi (0)+\psi (t) \frac{e^{\sigma t}-1}{\sigma } \le \frac{K_2|\Omega |}{\sigma }\left( -t+\frac{e^{\sigma t}-1}{\sigma }\right) . \end{aligned}$$

Since $\sigma \phi (0)-K_2|\Omega | t \le \sum _{i=1}^m c_i \int _{\Omega }u_{i 0}(x)\,d x$ (see (3.2)) it follows that

$$\begin{aligned} \psi (t) \le \left( e^{\sigma t}-1\right) ^{-1} \sum _{i=1}^m c_i \int _{\Omega } u_{i, 0}(x) d x+K_2|\Omega | \sigma ^{-1}, \end{aligned}$$

which finishes the proof of Lemma 3.1. $\square$

### Proof of Theorem 1.1-uniform-in-time boundness

We first show that for any $1\le p<\infty$, there exists a constant $C_p>0$ such that

3.3 $$\begin{aligned} \sup _{t\ge 0}\Vert u_i(t)\Vert _{L^{p}(\Omega )}\le C_p, \quad \forall i=1,\ldots ,m. \end{aligned}$$

Indeed, by using the $L^p$-energy function $\mathscr {L}_p[u]$ defined in (2.2) and similar to Lemma 2.8, we obtain

$$\begin{aligned} \begin{aligned} \mathscr {L}_p^{\prime }(t)+&\alpha _p \sum _{k=1}^m \int _{\Omega }u_k(x,t)^{b+p-2} \left| \nabla u_k(x, t)\right| ^2 \,dx \\ {}&\le C\left( \int _{\Omega }\sum _{i=1}^{m}u_i(x,t)^{p-1+r}\,dx+1\right) . \end{aligned} \end{aligned}$$

Now, we apply Lemma 2.6 to the right hand side, and it is note that the $L^1$-bound is uniform in time (due to Lemma 3.1), we get

$$\begin{aligned} \begin{aligned} \mathscr {L}_p^{\prime }(t)+&\frac{\alpha _p}{2} \sum _{k=1}^m \int _{\Omega }\left( u_k(x,t)^{b+p-2} \left| \nabla u_k(x, t)\right| ^2 +u_k(x,t)^{b+p}\right) \,dx \le C(p). \end{aligned} \end{aligned}$$

This implies

$$\begin{aligned} \begin{aligned} \mathscr {L}_p^{\prime }(t) + C\mathscr {L}_p(t) \le C(p). \end{aligned} \end{aligned}$$

Thus, we have

$$\begin{aligned} \sup _{t\ge 0}\Vert u_i(t)\Vert _{L^p(\Omega )} \le C_{p} \quad \forall i=1, \ldots , m. \end{aligned}$$

Next we will show that the solution is bounded uniformly in time in sup norm. We use a smooth time-truncated function $\psi : \mathbb {R}\rightarrow [0,1]$ with

$$\begin{aligned} \psi (s)= {\left\{ \begin{array}{ll} 0, &{}s\le 0\\ 1, &{}s\ge 1 \end{array}\right. } \end{aligned}$$

and $0\le \phi '\le C$, and its shifted version $\psi _{\tau }(\cdot )=\psi (\cdot ,-\tau )$ for any $\tau \in \mathbb {N}$. Let $\tau \in \mathbb {N}$ be arbitrary. It is straightforward to show that since $u=(u_i)_{i=1,\ldots ,m}$ is a weak solution to (1.2), the function $\psi _{\tau }u=(\psi _{\tau }u_i)_{i=1,\ldots ,m}$ is a weak solution to the following

$$\begin{aligned} {\left\{ \begin{array}{ll} \partial _t (\psi _{\tau }u_i)-\nabla \cdot (D_i(x,t) \Phi (\psi _{\tau }u) \nabla (\psi _{\tau }u_i))=\psi '_{\tau }u_i+\psi _{\tau }f_i(u), &{}\Omega \times (\tau ,\tau +2)\\ (D_i(x,t) \Phi (\psi _{\tau }u) \nabla (\psi _{\tau }u_i))\cdot \nu =0, &{} \partial \Omega \times (\tau ,\tau +2)\\ (\psi _{\tau }u_i)(x,\tau )=0, &{}x\in \Omega . \end{array}\right. } \end{aligned}$$

Thanks to (3.3) and the polynomial growth (A5), we have

$$\begin{aligned} \psi _{\tau }' u_i+\psi _{\tau }f_i(u)\le G_i(x,t,u):=C(1+\sum ^m_{k=1} u_k^l)\in L^p(\Omega \times (\tau ,\tau +2)), \end{aligned}$$

where $1\le p<\infty$. Therefore, similar to the proof of Lemma 2.9, we get

$$\begin{aligned} \Vert \psi _{\tau }u_i\Vert _{L^{\infty }(\Omega \times (\tau ,\tau +2)}\le C, \quad \forall i=1,\ldots ,m, \end{aligned}$$

where C is a constant independent of $\tau \in \mathbb {N}$. Thanks to $\psi _{\tau }\ge 0$ and $\psi |_{(\tau +1,\tau +2)}\equiv 1$, we obtain finally the uniform-in-time bound

$$\begin{aligned} \sup _{\tau \in \mathbb {N}}\Vert u_i\Vert _{L^\infty (\Omega \times (\tau ,\tau +1))}\le C,\quad \forall i=1,\ldots ,m, \end{aligned}$$

and the proof of Theorem 1.1 is complete. $\square$

## Proof of Theorems 1.3–1.9

### Proof of Theorem 1.3

For the global existence, we only need to show that for any $1\le p<\infty$ and any $T>0$, there exists $C_{T,p}>0$ such that

4.1 $$\begin{aligned} \sup _{t \in (0, T)}\Vert u_i^{\varepsilon }(t)\Vert _{L^p(\Omega )} \le C_{T,p}, \quad \forall i=1,\ldots ,m. \end{aligned}$$

The rest follows exactly as in Lemma 2.9. To show (4.1), we use the $L^p$-energy functions $\mathscr {L}_p(t)$ constructed in Lemma 2.8 until (2.14) we end up with

4.2 $$\begin{aligned} \begin{aligned}&\mathscr {L}_p^{\prime }(t)+\alpha _p \sum _{i=1}^m \int _{\Omega }\left( u_i^{\varepsilon }(x,t)^{b+p-2} \left| \nabla u^{\varepsilon }_i(x, t)\right| ^2 +u_i^{\varepsilon }(x,t)^{b+p}\right) \,dx\\ {}&\quad \quad \le C\left( \sum _{i=1}^{m}\int _{\Omega }\left( u_i^{\varepsilon }(x,t)^{p-1+r} + u_i^{\varepsilon }(x,t)^{b+p}\right) \,dx+1\right) . \end{aligned} \end{aligned}$$

Since (1.7) and (1.8), we apply Lemma 2.6 to estimate

$$\begin{aligned} \begin{aligned}&\int _{\Omega }\left( u_i^{\varepsilon }(x,t)^{p-1+r} + u_i^{\varepsilon }(x,t)^{b+p}\right) \,dx\\ {}&\le \frac{\alpha _p}{2}(\int _{\Omega } u_i^{\varepsilon }(x,t)^{p-2+b}|\nabla u_i^{\varepsilon }(x,t)|^{2}\,dx+\int _{\Omega } u_i^{\varepsilon }(x,t)^{b+p}\,dx)+C_{T}, \end{aligned} \end{aligned}$$

where $C_{T}$ depends on $\mathscr {F}(T)$. Inserting this into (4.2) yields

$$\begin{aligned} \begin{aligned} \mathscr {L}_p^{\prime }(t)+\sigma \mathscr {L}_p(t) \le C(T,p), \end{aligned} \end{aligned}$$

which implies (4.1).

For the uniform-in-time bounds, we use an argument similar to the proof of Theorem 1.1. $\square$

### Proof of Theorem 1.5

For the global existence, we only need to show that for any $1\le p<\infty$ and any $T>0$, there exists $C_{T,p}>0$ such that

4.3 $$\begin{aligned} \sup _{t \in (0, T)}\Vert u_i^{\varepsilon }(t)\Vert _{L^p(\Omega )} \le C_{T,p}, \quad \forall i=1,\ldots ,m. \end{aligned}$$

The rest follows exactly as in Lemma 2.9. To show (4.3), we use the $L^p$-energy functions $\mathscr {L}_p(t)$ constructed in Lemma 2.8 until (2.14) we obtain

4.4 $$\begin{aligned} \begin{aligned} \mathscr {L}_p^{\prime }(t)+&\frac{\alpha _p}{2} \sum _{i=1}^m \int _{\Omega }\left( u_i^{\varepsilon }(x,t)^{b+p-2} \left| \nabla u^{\varepsilon }_i(x, t)\right| ^2 +u_i^{\varepsilon }(x,t)^{b+p}\right) \,dx\\ {}&\le C\left( \sum _{i=1}^{m}\int _{\Omega }u_i^{\varepsilon }(x,t)^{p-1+r} \,dx+1\right) . \end{aligned} \end{aligned}$$

We integrate (4.4) in time to obtain

4.5 $$\begin{aligned} \begin{aligned} \sup _{t\in (0,T)}\mathscr {L}_p(t)+&\frac{\alpha _p}{2} \sum _{i=1}^m \int ^T_0\int _{\Omega }\left( u_i^{\varepsilon }(x,t)^{b+p-2} \left| \nabla u^{\varepsilon }_i(x, t)\right| ^2 +u_i^{\varepsilon }(x,t)^{b+p}\right) \,dxdt\\ {}&\le C\sum _{i=1}^{m}\int ^T_0\int _{\Omega }u_i^{\varepsilon }(x,t)^{p-1+r} \,dxdt+C_{p,T}+\mathscr {L}_p(0). \end{aligned} \end{aligned}$$

Denote by $y_i:=u_i^{\varepsilon }(x,t)^{\frac{b+p}{2}}$. The left-hand side (LHS) of (4.5) can be estimated below by

4.6 $$\begin{aligned} \begin{aligned} C\sum _{i=1}^{m}\left( \Vert y_i\Vert ^{\frac{2p}{b+p}}_{L^{\infty }(0,T; L^{\frac{2p}{b+p}}(\Omega ))}+\Vert y_i\Vert ^2_{L^2(0,T; H^1(\Omega ))} \right) \le \text {(LHS) of } (4.5). \end{aligned} \end{aligned}$$

For the right-hand side of (4.5), we first consider

4.7 $$\begin{aligned} \begin{aligned} \vartheta >p-1+r \end{aligned} \end{aligned}$$

as a constant to be determined later. Of course we are only interested in the case when $p-1+r>q$, otherwise the right hand side of (4.5) is bounded thanks to (1.10). By the interpolation inequality we have

4.8 $$\begin{aligned} \begin{aligned} \int _0^T\int _{\Omega }u^{\varepsilon }_i(x,t)^{p-1+r}dxdt&= \Vert u^{\varepsilon }_i(x,t)\Vert _{L^{p-1+r}(Q_T)}^{p-1+r}\\ {}&\le \Vert u^{\varepsilon }_i(x,t)\Vert _{L^{q}(Q_T)}^{\theta (p-1+r)}\Vert u^{\varepsilon }_i(x,t)\Vert _{L^{\vartheta }(Q_T)}^{(1-\theta )(p-1+r)}, \end{aligned} \end{aligned}$$

where $$\theta \in (0,1)$$ satisfies

$$\begin{aligned} \frac{1}{p-1+r} = \frac{\theta }{q} + \frac{1-\theta }{\vartheta }, \end{aligned}$$

which implies

$$\begin{aligned} (1-\theta )(p-1+r) = \frac{\vartheta (p-1+r-q)}{\vartheta - q}. \end{aligned}$$

Using this, and taking into account (1.10), (4.8) implies

4.9 $$\begin{aligned} \begin{aligned} \int _0^T\int _{\Omega }u^{\varepsilon }_i(x,t)^{p-1+r}dxdt&\le \mathscr {F}(T)^{\theta (p-1+r)}\Vert u^{\varepsilon }_i(x,t)\Vert _{L^{\vartheta }(Q_T)}^{\frac{\vartheta (p-1+r-q)}{\vartheta - q}}\\ {}&= \mathscr {F}(T)^{\theta (p-1+r)}\left( \int _0^T\int _{\Omega }u^{\varepsilon }_i(x,t)^{\vartheta }dxdt\right) ^{\frac{p-1+r-q}{\vartheta -q}}\\ {}&= C_T\left( \int _0^T\int _{\Omega }y_i^{\frac{2\vartheta }{b+p}}dxdt\right) ^{\frac{p-1+r-q}{\vartheta - q}}. \end{aligned} \end{aligned}$$

For *p* large enough, we choose $\vartheta$ close enough to (but bigger than) $p-1+r$ so that $H^1(\Omega )\hookrightarrow L^{\frac{2\vartheta }{b+p}}(\Omega )$. That means $\vartheta$ is arbitrary for $N\le 2$ and

4.10 $$\begin{aligned} \frac{2\vartheta }{b+p} \le \frac{2N}{N-2} \Leftrightarrow \vartheta \le \frac{(b+p) N}{N-2} \quad \text { for }\quad N\ge 3. \end{aligned}$$

Thus, we can use the Gagliardo-Nirenberg’s inequality to estimate

4.11 $$\begin{aligned} \int _{\Omega }y_i^{\frac{2\vartheta }{b+p}}dx = \Vert y_i\Vert _{L^{\frac{2\vartheta }{b+p}}(\Omega )}^{\frac{2\vartheta }{b+p}} \le C\Vert y_i\Vert _{H^1(\Omega )}^{\alpha \cdot \frac{2\vartheta }{b+p}}\Vert y_i\Vert _{L^{\frac{2p}{b+p}}(\Omega )}^{(1-\alpha )\cdot \frac{2\vartheta }{b+p}} \end{aligned}$$

where $$\alpha \in (0,1)$$ satisfies

$$\begin{aligned} \frac{b+p}{2\vartheta } = \left( \frac{1}{2} - \frac{1}{N}\right) \alpha + \frac{(1-\alpha )(b+p)}{2p}. \end{aligned}$$

From this

$$\begin{aligned} \alpha \cdot \frac{2\vartheta }{b+p} = \frac{2N(\vartheta -p)}{Nb+2p} \quad \text { and } \quad (1-\alpha )\cdot \frac{2\vartheta }{b+p} = \frac{2Np(b+p) - 2(N-2)p\vartheta }{(b+p)(Nb+2p)}. \end{aligned}$$

Therefore, we obtain from (4.11) that

$$\begin{aligned} \int _{\Omega }y_i^{\frac{2\vartheta }{b+p}}dx \le C\Vert y_i\Vert _{H^1(\Omega )}^{\frac{2N(\vartheta -p)}{Nb+2p}} \Vert y_i\Vert _{L^{\frac{2p}{b+p}}(\Omega )}^\frac{2Np(b+p) - 2(N-2)p\vartheta }{(b+p)(Nb+2p)}. \end{aligned}$$

It follows that

$$\begin{aligned} \int _0^T\int _{\Omega }y_i^{\frac{2\vartheta }{b+p}}dxdt&\le C\int _0^T\Vert y_i\Vert _{H^1(\Omega )}^{\frac{2N(\vartheta -p)}{Nb+2p}} \Vert y_i\Vert _{L^{\frac{2p}{b+p}}(\Omega )}^{\frac{2Np(b+p) - 2(N-2)p\vartheta }{(b+p)(Nb+2p)}} \,dt\\ {}&\le C\Vert y_i\Vert _{L^{\infty }(0,T;L^{\frac{2p}{b+p}}(\Omega ))}^{\frac{2Np(b+p) - 2(N-2)p\vartheta }{(b+p)(Nb+2p)}} \int _0^T\Vert y_i\Vert _{H^1(\Omega )}^{\frac{2N(\vartheta -p)}{Nb+2p}} \,dt. \end{aligned}$$

We choose

4.12 $$\begin{aligned} \frac{2N(\vartheta -p)}{Nb+2p} \le 2 \Leftrightarrow \vartheta \le \frac{Np+Nb+2p}{N}=b+p+\frac{2p}{N}, \end{aligned}$$

which is possible since $p - 1 + r < p + b+ \frac{2p}{N}$ for *p* large enough. Thus, by Hölder’s inequality,

$$\begin{aligned} \int _0^T\int _{\Omega }y_i^{\frac{2\vartheta }{b+p}}dxdt \le C_T\Vert y_i\Vert _{L^{\infty }(0,T;L^{\frac{2p}{b+p}}(\Omega ))}^{\frac{2Np(b+p) - 2(N-2)p\vartheta }{(b+p)(Nb+2p)}} \Vert y_i\Vert _{L^2(0,T;H^1(\Omega ))}^{\frac{2N(\vartheta -p)}{Nb+2p}}. \end{aligned}$$

Inserting this into (4.9) yields

4.13 $$\begin{aligned} \begin{aligned} \int _0^T\int _{\Omega }&u_i^{\varepsilon }(x,t)^{p-1+r}dxdt \le C_T\left( \Vert y_i\Vert _{L^{\infty }(0,T;L^{\frac{2p}{b+p}}(\Omega ))}^{\frac{2Np(b+p) - 2(N-2)p\vartheta }{(b+p)(Nb+2p)}} \Vert y_i\Vert _{L^2(0,T;H^1(\Omega ))}^{\frac{2N(\vartheta -p)}{Nb+2p}}\right) ^{\frac{p-1+r-q}{\vartheta - q}} \end{aligned} \end{aligned}$$

where $$\theta \in (0,1).$$

Using Young’s inequality of the form

$$\begin{aligned} x^{\lambda _1}y^{\lambda _2} \le \varepsilon (x^{\frac{2p}{b+p}}+y^2) + C_{\varepsilon } \quad \text { for } \quad \lambda _1\frac{b+p}{2p}+\frac{\lambda _2}{2} < 1, \end{aligned}$$

we can choose $\vartheta$ such that

4.14 $$\begin{aligned} \begin{aligned} \frac{N(b+p) - (N-2)\vartheta }{(Nb+2p)}\frac{p-1+r-q}{\vartheta - q} + \frac{N(p-1+r-q)(\vartheta - p)}{(Nb+2p)(\vartheta - q)}<1. \end{aligned}\nonumber \\ \end{aligned}$$

This is equivalent to

4.15 $$\begin{aligned} \vartheta > \frac{Nb(p-1+r)+2pq}{Nb+2(1+q-r)}. \end{aligned}$$

We now check that we can choose $\vartheta$ which satisfies all the conditions (4.7), (4.10), (4.12) and (4.15). This is fulfilled provided

$$\begin{aligned} \frac{Nb(p-1+r)+2pq}{Nb+2(1+q-r)}<b+p+\frac{2p}{N}, \;\text {which is equivalent to}\; r < 1 + \frac{N}{N+2}b + \frac{2q}{N+2}, \end{aligned}$$

and this can be obtain from our assumption (1.11). We estimate (4.13) further as

$$\begin{aligned} \int _0^T\int _{\Omega }u_i^{\varepsilon }(x,t)^{p-1+r}dxdt \le \varepsilon \left( \Vert y_i\Vert _{L^{\infty }(0,T;L^{\frac{2p}{b+p}}(\Omega ))}^{\frac{2p}{b+p}} + \Vert y_i\Vert _{L^2(0,T;H^1(\Omega ))}^2\right) + C_{T,\varepsilon }. \end{aligned}$$

Thus

$$\begin{aligned} \text {RHS of} (4.5) \le \mathscr {L}_p(0) + \varepsilon \sum _{i=1}^{m}\left( \Vert y_i\Vert _{L^{\infty }(0,T;L^{\frac{2p}{b+p}}(\Omega ))}^{\frac{2p}{b+p}} + \Vert y_i\Vert ^2_{L^2(0,T;H^1(\Omega ))}\right) + C_{T,\varepsilon }. \end{aligned}$$

Combining this with (4.6) gives us the desired estimate (4.3).

For the uniform-in-time bounds, We use an argument similar to the proof of Theorem 1.1. $\square$

### Proof of Theorem 1.7

We give a formal proof as its rigor can be easily obtained through approximation. Since $h_k(\cdot )$ is convex, we have

$$\begin{aligned} \nabla \cdot (D_i\Phi (u)\nabla (h_i(u_i)))&= \nabla \cdot (D_i\Phi (u)h_i^{\prime }(u_i)\nabla u_i)\\ {}&\ge h'(u_i)\nabla \cdot (D_i\Phi (u)\nabla u_i) + \lambda h_i''(u_i)\Phi (u)|\nabla u_i|^2\\ {}&\ge h'(u_i)\nabla \cdot (D_i\Phi (u)\nabla u_i). \end{aligned}$$

Therefore, by defining $v_i:= h_i(u_i)\ge 0$ we have

4.16 $$\begin{aligned} \partial _t v_i - \nabla \cdot (D_i\Phi (u)\nabla v_i) \le G_i(x,t,u):= h_i^{\prime }(u_i)f_i(x,t,u), \quad x\in \Omega , \; t>0, \end{aligned}$$

with initial data $v_i(x,0) = h_i(u_{i,0}(x))$ and homogeneous Dirichlet boundary condition $v_i(x,t) = 0$ for $x\in \partial \Omega$ and $t>0$. Thanks to (H1) and we have

$$\begin{aligned} \sum _{i=1}^{m}G_i(x,t,u) \le K_5\sum _{i=1}^{m}v_i + K_6 \end{aligned}$$

and

$$\begin{aligned} A\begin{pmatrix}G_i(x,t,u)\\ \cdots \\ G_m(x,t,u)\end{pmatrix} \le K_7\overrightarrow{1}\left( \sum _{i=1}^{m}v_i + 1\right) ^r. \end{aligned}$$

We can now reapply the methods in the proof of Theorem 1.1 to obtain $v_i \in L^\infty _{\text {loc}}(0,\infty ;L^{\infty }(\Omega ))$, and in case $K_5<0$ or $K_5 = K_6 = 0$ in (H1),

$$\begin{aligned} \text {ess}\sup _{t\ge 0}\Vert v_i(t)\Vert _{L^{\infty }(\Omega )} <+\infty , \quad \forall i=1,\ldots , m. \end{aligned}$$

Due to the assumption (H1), the global existence and boundedness of (1.2) immediately hold. $\square$

### Definition 4.1

A vector of non-negative concentrations $u = (u_1, \ldots , u_m)$ is called a weak solution to (1.15) on (0, *T*) if

$$\begin{aligned} u_i\in C([0,T]; L^{2}(\Omega )), ~~ \Phi (u)\nabla u_i\in L^2(0,T;L^2(\Omega )), \quad f_i(u)\in L^2(0,T;L^{2}(\Omega )), \end{aligned}$$

with $u_i(\cdot ,0)= u_{i,0}(\cdot )$ for all $i=1,\ldots , m$, and for any test function $\varphi \in L^2(0,T;H^1(\Omega ))$ with $\partial _t\varphi \in L^2(0,T;H^{-1}(\Omega ))$, it holds that

4.17 $$\begin{aligned} \begin{aligned} \int _{\Omega }u_i(x,t)\varphi (x,\cdot )dx\bigg |_{0}^{T} - \int _0^T\int _{\Omega }u_i\partial _t\varphi \,dxdt + \int _0^T\int _{\Omega }D_i(x,t)\Phi (u)\nabla u_i \cdot \nabla \varphi \,dxdt\\ +\alpha _i\int _0^T\int _{\partial \Omega }u_i\varphi d\mathscr {H}^{n-1} \,dt = \int _0^T\int _{\Omega }f_i(x,t,u)\varphi \,dxdt. \end{aligned}\nonumber \\ \end{aligned}$$

### Proof of Theorem 1.9

The proof of this theorem is similar to that of Theorems 1.1, 1.3 and 1.5, except for the fact that the $L^1$-norm can be obtained in a different, and easier, way. Since $\varphi \equiv 1$ is an admissible test function we get from (4.17) and (A3) that

$$\begin{aligned} \sum _{i=1}^{m}\int _{\Omega }c_iu_i(\cdot ,t) \,dx\bigg |_{t=0}^{t=T} + \sum _{i=1}^{m}c_i\alpha _i\int _0^T\int _{\partial \Omega } u_id\mathscr {H}^{n-1} \,dt\\ \le K_1\int _0^T\int _{\Omega }u_i \,dxdt + K_2|\Omega |T. \end{aligned}$$

The $L^1$-bound is uniform in time in case $K_1<0$ or $K_1 = K_2 =0$ (using a similar idea to Lemma 3.1). $\square$

## Applications

We show the application of our results to SEIR(-D) model considered in [1, 31, 32] and its variants. First, the SEIR model introduced in [1, 31, 32] reads as

5.1 $$\begin{aligned} {\left\{ \begin{array}{ll} \partial _t s= \alpha n - (1 - A_0/n) \beta _i si - (1 - A_0/n) \beta _e se - \mu s + \nabla \cdot (n \nu _s \nabla s),\\ \partial _t e= (1 - A_0/n) \beta _i si + (1 - A_0/n) \beta _e se - \sigma e - \phi _e e - \mu e + \nabla \cdot (n \nu _e \nabla e), \\ \partial _t i = \sigma e - \phi _d ni - \phi _r i - \mu i + \nabla \cdot (n \nu _i \nabla i), \\ \partial _t r = \phi _r i + \phi _e e - \mu r + \nabla \cdot (n \nu _r \nabla r), \end{array}\right. }\nonumber \\ \end{aligned}$$

subject to homogeneous Neumann boundary conditions $\nabla s \cdot \eta =\nabla e \cdot \eta =\nabla i \cdot \eta =\nabla r \cdot \eta =0$ on $\partial \Omega$, where the symbols *s*, *e*, *i* and *r* denote the susceptible-, exposed-, infected-, recovered populations, respectively, and $n:=s+e+i+r$ is the total living population, $\nu _s$, $\nu _e$, $\nu _i$, $\nu _r$, $\beta _i$, $\beta _e$, $\alpha$, $\mu$, $\phi$, $\sigma$, $\phi _d$, $\phi _r$ and $\phi _e$ are positive constants. The global existence of bounded weak solutions was shown in [1] where it was imposed that all diffusion rates are the same, i.e. $\nu _s = \nu _e = \nu _i = \nu _r$, the term $1-A_0/n$ is replaced by a non-singular function *A*(*n*), for instance $A(n) = (1-A_0/n)_+$, and the term $-\phi _dni$ is replaced by $-\phi _d i$. These are needed to show the total population *n* is pointwise bounded from below by a positive constant, making the diffusion operators non-degenerate.^3^ The method therein seems to break down when the diffusion are different. By applying our main results in Theorem 1.1, we show that these assumptions can be removed. We have the following result.

### Theorem 5.1

Assume all diffusion coefficients $\nu _s, \nu _e, \nu _i, \nu _r$, and other parameters in (5.1) to be positive. Then, for any non-negative and bounded initial data $(s_0,e_0,i_0,r_0)\in L_+^{\infty }(\Omega )^4$, there is a global bounded weak solution to (1.1), i.e. for any $T>0$,

5.2 $$\begin{aligned} \sup _{t\in (0,T)}\left( \Vert s(t)\Vert _{L^{\infty }(\Omega )}+\Vert e(t)\Vert _{L^{\infty }(\Omega )}+ \Vert i(t)\Vert _{L^{\infty }(\Omega )}+ \Vert r(t)\Vert _{L^{\infty }(\Omega )} \right) <\infty . \end{aligned}$$

Moreover, if $\alpha \le \mu$, then this solution is bounded uniformly in time, i.e.

5.3 $$\begin{aligned} \sup _{t>0}\big (\Vert s(t)\Vert _{L^{\infty }(\Omega )}+ \Vert e(t)\Vert _{L^{\infty }(\Omega )}+ \Vert i(t)\Vert _{L^{\infty }(\Omega )}+ \Vert r(t)\Vert _{L^{\infty }(\Omega )} \big ) < +\infty . \end{aligned}$$

### Proof

It is noted that the nonlinearities in (5.1) are not locally Lipschitz continuous around 0. We circumvent this problem by considering the following approximating system for each $\delta >0$,

5.4 $$\begin{aligned} {\left\{ \begin{array}{ll} \partial _t s^{\delta }&{}= \alpha n^{\delta } - (1 - \frac{A_0}{n^{\delta } + \delta } ) \beta _i s^{\delta } i^{\delta } - (1 - \frac{A_0}{n^{\delta } + \delta } ) \beta _e s^{\delta } e^{\delta } - \mu s^{\delta }\\ &{}\qquad \quad + \nabla \cdot (n^{\delta } \nu _s \nabla s^{\delta }),\\ \partial _t e^{\delta }&{}= (1 - \frac{A_0}{n^{\delta } + \delta } ) \beta _i s^{\delta } i^{\delta } + (1 - \frac{A_0}{n^{\delta } + \delta } ) \beta _e s^{\delta } e^{\delta } - \sigma e^{\delta } - \phi _e e^{\delta }\\ {} &{}\qquad \, - \mu e^{\delta } + \nabla \cdot (n^{\delta } \nu _e \nabla e^{\delta }),\\ \partial _t i^{\delta } &{}= \sigma e^{\delta } - \phi _d n^{\delta } i^{\delta } - \phi _r i^{\delta } - \mu i^{\delta } + \nabla \cdot (n^{\delta } \nu _i \nabla i^{\delta }), \\ \partial _t r^{\delta } &{}= \phi _r i^{\delta } + \phi _e e^{\delta } - \mu r^{\delta } + \nabla \cdot (n^{\delta } \nu _r \nabla r^{\delta }), \end{array}\right. } \end{aligned}$$

subject to homogeneous Neumann boundary conditions $\nabla s^{\delta } \cdot \eta =\nabla e^{\delta } \cdot \eta =\nabla i^{\delta } \cdot \eta =\nabla r^{\delta } \cdot \eta =0$ on $\partial \Omega$, where $u^\delta = (s^\delta , e^\delta , i^\delta , r^\delta )$, $n^\delta = s^\delta + e^\delta + i^\delta + r^\delta$, $f^{\delta }_s, f^{\delta }_e, f^{\delta }_i$ and $f^{\delta }_r$ the nonlinearities in the equations of $s^{\delta }, e^{\delta }, i^{\delta }$ and $r^{\delta }$, respectively. Consequently

$$\begin{aligned} {\left\{ \begin{array}{ll} f^{\delta }_s(u^{\delta }) \le \alpha n^{\delta } + (A_0\beta _i + A_0\beta _e)s^{\delta } \\ f^{\delta }_s(u^{\delta }) + f^{\delta }_e(u^{\delta })\le \alpha n^{\delta }\\ f^{\delta }_s(u^{\delta }) + f^{\delta }_e(u^{\delta }) + f^{\delta }_i(u^{\delta }) \le \alpha n^{\delta }\\ f^{\delta }_s(u^{\delta }) + f^{\delta }_e(u^{\delta }) + f^{\delta }_i(u^{\delta }) + f^{\delta }_r(u^{\delta }) \le (\alpha - \mu ) n^{\delta }. \end{array}\right. } \end{aligned}$$

Similar to the proof of Theorem 1.1, we can get

5.5 $$\begin{aligned} \Vert s^{\delta }\Vert _{L^{\infty }(Q_T)}, \Vert e^{\delta }\Vert _{L^{\infty }(Q_T)}, \Vert i^{\delta }\Vert _{L^{\infty }(Q_T)}, \Vert r^{\delta }\Vert _{L^{\infty }(Q_T)} \le C_T, \end{aligned}$$

where $C_T$ independent of $\delta$. This independence on $\delta$ can be obtained since the constant $C_T$ obtained in Theorem 1.1 depends only on the size of initial data, domain $\Omega$, diffusion coefficients, and the parameters in the assumptions (A1)–(A5). Due to the polynomial growth of $f^{\delta }_s, f^{\delta }_e, f^{\delta }_i$ and $f^{\delta }_r$, we can get

$$\begin{aligned} \Vert f^{\delta }_s (u^{\delta })\Vert _{L^{\infty }(Q_T)}, \Vert f^{\delta }_e (u^{\delta })\Vert _{L^{\infty }(Q_T)}, \Vert f^{\delta }_i (u^{\delta })\Vert _{L^{\infty }(Q_T)}, \Vert f^{\delta }_r (u^{\delta })\Vert _{L^{\infty }(Q_T)} \le C_T. \end{aligned}$$

By multiply the first equation of (5.4) by $s^{\delta }$ then integrating on $\Omega _T$ gives

5.6 $$\begin{aligned} \sup _{t\in (0,T)}\int _{\Omega }s^{\delta }(t)^2 + \nu _s \int _0^T\int _{\Omega }n^{\delta }|\nabla s^{\delta }|^{2}dxdt \le C_T. \end{aligned}$$

Since $n^\delta \ge s^\delta$, it follows

5.7 $$\begin{aligned} C_T \ge \int _0^T\int _{\Omega }s^\delta |\nabla s^\delta |^2dxdt \ge C \Vert \nabla (s^{\delta })^{\frac{3}{2}} \Vert _{L^{2}(Q_T)}^2. \end{aligned}$$

By testing the first equation of (5.4) with $\phi \in L^2(0,T; H^1(\Omega ))$, we have

$$\begin{aligned}&\int _0^T\int _{\Omega }\partial _t s^{\delta } \phi \,dxdt = - \nu _s\int _0^T\int _{\Omega }n^{\delta } \nabla s^{\delta } \cdot \nabla \phi \,dxdt + \int _0^T\int _{\Omega }f^{\delta }_s(u^{\delta }) \phi \,dxdt\\ {}&\qquad \le \nu _s\Vert \sqrt{n^\delta }\Vert _{L^{\infty }(Q_T)}\Vert \sqrt{n^\delta }|\nabla s^\delta |\Vert _{L^{2}(Q_T)}\Vert \nabla \phi \Vert _{L^{2}(Q_T)} + \Vert f_s^{\delta }(u^\delta )\Vert _{L^{2}(Q_T)}\Vert \phi \Vert _{L^{2}(Q_T)}\\&\qquad \le C_T \Vert \phi \Vert _{L^2(0,T; H^1(\Omega ))}, \end{aligned}$$

where we used (5.5) and (5.6). Thus,

$$\begin{aligned} \Vert \partial _t s^{\delta }\Vert _{L^2(0, T;(H^1(\Omega ))')} \le C_T. \end{aligned}$$

Applying a nonlinear version of Aubin-Lions Lemma, see e.g. [21, Theorem 1] to ensure

5.8 $$\begin{aligned} s^{\delta }{\mathop {\longrightarrow }\limits ^{\delta \rightarrow 0}} s \quad \text {strongly in } L^{2}(Q_T) \end{aligned}$$

Thanks to the $L^{\infty }$ bound of $s^{\delta }$, this convergence in fact holds in $L^{p}(Q_T)$ for any $1\le p<\infty$. From the inequality (5.7), the sequence $(s^{\delta })^{\frac{3}{2}}$ is uniformly bounded in $L^2(0,T; H^{1}(\Omega ))$, there exists a subsequence (still denoted by $(s^{\delta })$) and $v\in L^2(0,T; H^{1}(\Omega ))$ such that

$$\begin{aligned} (s^{\delta })^{\frac{3}{2}} \rightharpoonup v \quad \text {weekly in } L^2(0,T; H^{1}(\Omega )). \end{aligned}$$

Now, we are ready to identify the weak limit *v* by the following computation: for testing functions $\varphi \in C^{\infty }_0(Q_T)$ we have

$$\begin{aligned} \int _0^T\int _{\Omega }\nabla v \cdot \varphi \,dxdt&= \lim _{\delta \rightarrow 0} \int _0^T\int _{\Omega }\nabla (s^{\delta })^{\frac{3}{2}} \cdot \varphi \,dxdt\\ {}&= - \lim _{\delta \rightarrow 0} \int _0^T\int _{\Omega }(s^{\delta })^{\frac{3}{2}} \cdot \nabla \varphi \,dxdt\\ {}&= - \int _0^T\int _{\Omega }s^{\frac{3}{2}} \cdot \nabla \varphi \,dxdt. \end{aligned}$$

This implies that the function $s^{\frac{3}{2}}$ is weakly differentiable with weak derivative *v*, i.e., $s^{\frac{3}{2}} = v$. Thus

5.9 $$\begin{aligned} (s^{\delta })^{\frac{3}{2}} \rightharpoonup s^{\frac{3}{2}} \quad \text {weekly in } L^2(0,T; H^{1}(\Omega )). \end{aligned}$$

Similarly, we can get the strong convergence of $(e^{\delta }, i^{\delta }, r^{\delta })$ in $L^{p}(Q_T)^3$ for any $1\le p<\infty$, and the weak convergence of $((e^{\delta })^{\frac{3}{2}}, (i^{\delta })^{\frac{3}{2}}, (r^{\delta })^{\frac{3}{2}})$ in $L^2(0, T; H^1(\Omega ))^3$. Thus, $n^{\delta } = s^\delta + e^\delta + i^\delta + r^\delta$ converges strongly to *n* in $L^{p}(Q_T)$ for any $1\le p<\infty$. Now, let $\delta \rightarrow 0$ in the weak formulation of (5.4) with $\psi \in L^2(0, T; H^1(\Omega ))$ and using [7, Lemma A.2], we get the weak solution $u= (s, e, i, r)$ of (5.1).

Furthermore, if $\alpha \le \mu$, we have the uniform-in-time boundedness first for solutions to (5.4) and consequently for (5.1). $\square$

Thanks to the robustness of our approach, we can also consider a variant (5.1) where

- $\beta _i$ and $\beta _e$ are functions of *n* or the combinations of *n* and species *i* or *e* (see the original model proposed in [32]);
- the diffusion rates $\nu _s, \nu _e, \nu _i, \nu _r$ are functions of (*x*, *t*), which shows the possibly high heterogeneity of the environment; and
- and the other rates $\alpha , \mu , \sigma , \phi _e, \phi _d, \phi _r$ are non-negative functions of (*x*, *t*),

and therefore the system reads as

5.10 $$\begin{aligned} \left\{ \begin{aligned} \partial _t s&= \alpha n - (1-\frac{A_0}{n}) \beta _i(i,n) si - (1-\frac{A_0}{n}) \beta _e(e,n) se\\ {}&\quad - \mu s + \nabla \cdot (n \nu _s \nabla s),\\ \partial _t e&= (1-\frac{A_0}{n}) \beta _i(i,n) si + (1-\frac{A_0}{n}) \beta _e(e,n) se - \sigma e\\ {}&\quad - \phi _e e - \mu e + \nabla \cdot (n \nu _e \nabla e),\\ \partial _t i&= \sigma e - \phi _dn i - \phi _r i - \mu i + \nabla \cdot (n \nu _i \nabla i),\\ \partial _t r&= \phi _r i + \phi _e e - \mu r + \nabla \cdot (n \nu _r \nabla r), \end{aligned} \right. \end{aligned}$$

subject to homogeneous Neumann boundary conditions $\nabla s \cdot \eta =\nabla e \cdot \eta =\nabla i \cdot \eta =\nabla r \cdot \eta =0$. We assume the following:

1. $\beta _i(i,n), \beta _e(e,n)$ are non-negative continuous functions and satisfy
  $$\begin{aligned} \beta _i(i,n) \le c_i \left( 1 + i + n\right) , \quad \beta _e(e,n) \le c_e\left( 1 + e+n\right) , \end{aligned}$$
  for some constants $c_i, c_e>0$,
2. for $z\in \{s,e,i,r\}$, $\nu _z: \Omega \times [0,\infty ) \rightarrow \mathbb {R}^{n\times n}$ such that $\nu _z\in L^\infty (\Omega \times (0,T);\mathbb {R}^{n\times n})$ for each $T>0$, and there is a constant $\lambda _z>0$ such that
  $$\begin{aligned} \lambda _z|\xi |^2 \le \xi ^\top \nu _z(x,t) \xi , \quad \forall (x,t)\in \Omega \times [0,\infty ), \quad \forall \xi \mathbb {R}^n; \end{aligned}$$
3. all non-negative functions $\alpha , \mu , \sigma , \phi _e, \phi _d, \phi _r: \Omega \times [0,\infty )$ are bounded by a common constant, i.e. $\exists M>0$ such that
  $$\begin{aligned} (\alpha + \mu + \sigma + \phi _e + \phi _d+\phi _r)(x,t) \le M, \quad \forall (x,t)\in \Omega \times [0,\infty ). \end{aligned}$$

The following theorem follows directly from Theorem 1.1.

### Theorem 5.2

Assume (M1’)–(M3’). Then, for any non-negative bounded initial data, there exists a global bounded weak solution to (5.10), i.e. for any $T>0$,

$$\begin{aligned} \sup _{t\in (0,T)}\big (\Vert s(t)\Vert _{L^{\infty }(\Omega )}+\Vert e(t)\Vert _{L^{\infty }(\Omega )}+ \Vert i(t)\Vert _{L^{\infty }(\Omega )}+\Vert r(t)\Vert _{L^{\infty }(\Omega )} \big ) < +\infty . \end{aligned}$$

In particular, if $\alpha (x,t)\le \mu (x,t)$ for all $(x,t)\in \Omega \times [0,\infty )$, then the solution is bounded uniformly in time, i.e.

$$\begin{aligned} \sup _{t>0}\big (\Vert s(t)\Vert _{L^{\infty }(\Omega )}+ \Vert e(t)\Vert _{L^{\infty }(\Omega )}+ \Vert i(t)\Vert _{L^{\infty }(\Omega )}+ \Vert r(t)\Vert _{L^{\infty }(\Omega )}\big ) < +\infty . \end{aligned}$$

## Data Availability

No datasets were generated or analysed during the current study.

## Proof of Lemma 2.9

We first state the following interpolation inequality.

### Lemma A.1

Let $\gamma \ge 1$ and $\alpha >0$. Then we have the following interpolation inequality

A.1 $$\begin{aligned} \Vert u\Vert ^{\lambda }_{L^{\sigma }(Q_T)}\le C \left( \Vert u\Vert ^{\gamma }_{L^{\infty }(0,T;L^{\gamma }(\Omega ))} + \Vert u\Vert ^{\alpha }_{L^{\alpha }(0,T;L^{\beta }(\Omega ))} \right) , \end{aligned}$$

where

A.2 $$\begin{aligned} {\left\{ \begin{array}{ll} \lambda =\frac{\alpha \beta +\gamma (\beta -\alpha )}{2\beta -\alpha } \quad \text {and} \quad \sigma =\frac{\alpha \beta +\gamma (\beta -\alpha )}{\beta }, &{}\text { if } \quad 0<\beta <\infty ,\\ \lambda =\frac{\alpha +\gamma }{2} \quad \text {and} \quad \sigma =\alpha +\gamma , &{}\text { if }\quad \beta =\infty . \end{array}\right. } \end{aligned}$$

### Proof

For $0<\beta <\infty$. We apply an interpolation inequality to get

A.3 $$\begin{aligned} \Vert u\Vert _{L^{\sigma }(Q_T)}\le \Vert u\Vert ^{\theta }_{L^{\infty }(0,T;L^{\gamma }(\Omega ))} \Vert u\Vert ^{1-\theta }_{L^{\alpha }(0,T;L^{\beta }(\Omega ))} \end{aligned}$$

with $$\theta \in (0,1)$$ satisfying

A.4 $$\begin{aligned} \frac{1}{\sigma }=\frac{\theta }{\infty } + \frac{1-\theta }{\alpha }=\frac{\theta }{\gamma } + \frac{1-\theta }{\beta }. \end{aligned}$$

It follows that

A.5 $$\begin{aligned} \theta =\frac{\gamma (\beta -\alpha )}{\alpha \beta +\gamma (\beta -\alpha )}, \quad 1-\theta =\frac{\alpha \beta }{\alpha \beta + \gamma (\beta -\alpha )} \quad \text {and} \quad \sigma =\frac{\alpha \beta + \gamma (\beta -\alpha )}{\beta }. \end{aligned}$$

From (A.3) and $\lambda >0$ to be chosen, using Young’s inequality we obtain

$$\begin{aligned} \begin{aligned} \Vert u\Vert ^{\lambda }_{L^{\sigma }(Q_T)}&\le \Vert u\Vert ^{\theta \lambda }_{L^{\infty }(0,T;L^{\gamma }(\Omega ))} \Vert u\Vert ^{(1-\theta )\lambda }_{L^{\alpha }(0,T;L^{\beta }(\Omega ))}\\ {}&\le \frac{\theta \lambda }{\gamma } \Vert u\Vert ^{\gamma }_{L^{\infty }(0,T;L^{\gamma }(\Omega ))} + \frac{\gamma -\theta \lambda }{\gamma } \Vert u\Vert ^{(1-\theta )\lambda \frac{\gamma }{\gamma -\theta \lambda }}_{L^{\alpha }(0,T;L^{\beta }(\Omega ))}. \end{aligned} \end{aligned}$$

Now, we choose $\lambda$ such that

$$\begin{aligned} \frac{(1-\theta ) \lambda \gamma }{\gamma -\theta \lambda }=\alpha , \end{aligned}$$

which is equivalent to

$$\begin{aligned} \lambda =\frac{\alpha \gamma }{(1-\theta )\gamma +\theta \alpha }=\frac{\alpha \beta +\gamma (\beta -\alpha )}{2\beta -\alpha }, \end{aligned}$$

where we used (A.5). Therefore

$$\begin{aligned} \Vert u\Vert ^{\lambda }_{L^{\sigma }(Q_T)}\le C \left( \Vert u\Vert ^{\gamma }_{L^{\infty }(0,T;L^{\gamma }(\Omega ))} + \Vert u\Vert ^{\alpha }_{L^{\alpha }(0,T;L^{\beta }(\Omega ))} \right) \end{aligned}$$

with

$$\begin{aligned} \lambda =\frac{\alpha \beta +\gamma (\beta -\alpha )}{2\beta -\alpha } \quad \text {and} \quad \sigma =\frac{\alpha \beta +\gamma (\beta -\alpha )}{\beta }. \end{aligned}$$

For $\beta =\infty$, we can use a similar method to get the result. This completes the proof of Lemma A.1. $\square$

### Lemma A.2

([10], Lemma 2.4) Let $\left\{ y_{n}\right\} _{n \ge 1}$ be a sequence of positive numbers which satisfies

$$\begin{aligned} y_{n+1} \le K B^{n}\left( y_{n}^{\gamma }+y_{n}^{\kappa }\right) \end{aligned}$$

where $K, B>0$ and $\gamma , \kappa >1$ are independent of *n*. Then there exists $\varepsilon >0$ such that, if $y_{1} \le \varepsilon$, then

$$\begin{aligned} \lim _{n \rightarrow \infty } y_{n}=0. \end{aligned}$$

We are now presenting a proof of Lemma 2.9 using the idea of Moser iteration.

### Proof of Lemma 2.9

From (A5),

$$\begin{aligned} f_i^{\varepsilon }(u^{\varepsilon })\le f_i(u^{\varepsilon })\le F_i(u^{\varepsilon }):=K_4\left( 1+\sum ^m_{j=1}(u_j^{\varepsilon })^l\right) . \end{aligned}$$

Using Lemma 2.8, we can get for any $1\le p<\infty$ a constant $C_p>0$ exists depending on *p* (but not on *T*) such that

A.6 $$\begin{aligned} \Vert f^{\varepsilon }_i(u^{\varepsilon })\Vert _{L^{p}(Q_T)}\le C_p, \quad \forall i=1,\ldots ,m. \end{aligned}$$

Let $k \ge 1$ be a constant which will be specified later. For each $j \ge 0$, we define

$$\begin{aligned} v_j:=\left( u^{\varepsilon }_i-k+\frac{k}{2^j}\right) _{+}=\max \left\{ u^{\varepsilon }_i-k+\frac{k}{2^j}, 0\right\} \end{aligned}$$

and

$$\begin{aligned} A_j:=\left\{ (x, t) \in Q_T: u^{\varepsilon }_i(x, t) \ge k-\frac{k}{2^j}\right\} . \end{aligned}$$

The following simple observations will be helpful

A.7 $$\begin{aligned} \begin{aligned}&v_{j+1}(x, t) \le v_j(x, t),{} & {} \forall (x, t) \in A_j, \\&v_j(x, t) \ge \frac{k}{2^{j+1}},{} & {} \forall (x, t) \in A_{j+1} \subset A_j. \end{aligned} \end{aligned}$$

By multiplying the equation $\partial _t u^{\varepsilon }_i - \Delta \left( D_i(x,t) \,\Phi (u^{\varepsilon })\, \nabla u^{\varepsilon }_i\right) =f^{\varepsilon }_i$ by $v_{j+1}$ and integrating on $Q_T$, we have

$$\begin{aligned} \begin{aligned}&\sup _{t \in (0, T)}\Vert v_{j+1}(t)\Vert ^2_{L^{2}(\Omega )} + 2 \lambda \int _0^T \int _{\Omega } \Phi (u^{\varepsilon }) \left| \nabla v_{j+1}\right| ^2 \,d x d t \\ {}&\quad \le \Vert v_{j+1}(0)\Vert ^2_{L^{2}(\Omega )} + 2 \int _0^T \int _{\Omega } f^{\varepsilon }_i v_{j+1} \,d x d t, \end{aligned} \end{aligned}$$

where we used (1.3). Using (Q2), we have

A.8 $$\begin{aligned} \begin{aligned}&\sup _{t \in (0, T)}\Vert v_{j+1}(t)\Vert ^2_{L^{2}(\Omega )} + 2 \lambda M_i \int _0^T \int _{\Omega }(u^{\varepsilon }_i)^{b}\left| \nabla v_{j+1}\right| ^2 \,d x d t \\ {}&\quad \le \Vert v_{j+1}(0)\Vert ^2_{L^{2}(\Omega )}+2 \int _0^T \int _{\Omega } f^{\varepsilon }_i v_{j+1} \,d x d t. \end{aligned} \end{aligned}$$

Note that $u^{\varepsilon }_i \ge k-\frac{k}{2^j} \ge \frac{k}{2}$ on $A_j$ ($j\ge 1$), we have

$$\begin{aligned} \begin{aligned} 2\lambda M_i \int _0^T \int _{\Omega }(u^{\varepsilon }_i)^{b}\left| \nabla v_{j+1}\right| ^2 \,d x d t&\ge 2\lambda M_i \iint _{A_j}(u^{\varepsilon }_i)^{b}\left| \nabla v_{j+1}\right| ^2 \,d x d t \\ {}&\ge 2\lambda M_i \left( \frac{k}{2}\right) ^{b} \iint _{A_j}\left| \nabla v_{j+1}\right| ^2 \,dx dt \\&\ge \frac{\lambda M_i}{2^{b-1}} \int _0^T \int _{\Omega }\left| \nabla v_{j+1}\right| ^2 \,dxdt, \end{aligned} \end{aligned}$$

thanks to $k\ge 1$ and the fact that $v_{j+1} \equiv 0$ on $Q_T \backslash A_{j+1} \supset Q_T \backslash A_j$ since $A_{j+1} \subset A_j$. By adding $\frac{\lambda M_i}{2^{b-1}} \int _0^T\Vert v_{j+1}\Vert ^2_{L^{2}(\Omega )} d t$ to both sides of (A.8), we get

$$\begin{aligned} \begin{aligned}&\sup _{t \in (0, T)}\Vert v_{j+1}(t)\Vert ^2_{L^{2}(\Omega )} + \frac{\lambda M_i}{2^{b-1}} \int _0^T\Vert v_{j+1}\Vert _{H^1(\Omega )}^2 \, d t \\ {}&\le \frac{\lambda M_i}{2^{b-1}} \int _0^T\Vert v_{j+1}\Vert ^2_{L^{2}(\Omega )}\, d t+\Vert v_{j+1}(0)\Vert ^2_{L^{2}(\Omega )} + 2\int _0^T \int _{\Omega } f^{\varepsilon }_i v_{j+1} \,d x d t, \end{aligned} \end{aligned}$$

which yields

A.9 $$\begin{aligned} \Vert v_{j+1}\Vert _{W(0, T)}^2 \le C\left( \int _0^T\Vert v_{j+1}\Vert ^2_{L^{2}(\Omega )} \, d t+\Vert v_{j+1}(0)\Vert ^2_{L^{2}(\Omega )} + \int _0^T \int _{\Omega } f^{\varepsilon }_i v_{j+1} \,d x d t \right) .\nonumber \\ \end{aligned}$$

By definition, when we choose $k \ge 2\Vert u^{\varepsilon }_0\Vert _{L^{\infty }(\Omega )}$, we have

A.10 $$\begin{aligned} \Vert v_{j+1}(0)\Vert ^2_{L^{2}(\Omega )}=\Vert \left( u^{\varepsilon }_0-k+\frac{k}{2^{j+1}}\right) _{+}\Vert ^2_{L^{2}(\Omega )}=0. \end{aligned}$$

By using (A.7), we have with $1 \le \frac{2^{j+1}}{k} v_j$ on $A_{j+1}$

A.11 $$\begin{aligned} \begin{aligned} \int _0^T \int _{\Omega }\left| v_{j+1}\right| ^2 d x d t&=\int _0^T \int _{\Omega } \textbf{1}_{A_{j+1}}\left| v_{j+1}\right| ^2 \,d x d t \\ {}&\le \int _0^T \int _{\Omega } \textbf{1}_{A_{j+1}}\left| v_j\right| ^2 \,d x d t \\ {}&\le \left( \frac{2^{j+1}}{k}\right) ^{\frac{4}{N}} \int _0^T \int _{\Omega } \textbf{1}_{A_{j+1}}\left| v_j\right| ^{2+\frac{4}{N}} \,d x d t \\ {}&\le C\left( 2^{\frac{4}{N}}\right) ^j\Vert v_j\Vert _{W(0, T)}^{2+\frac{4}{N}}. \end{aligned} \end{aligned}$$

Choose $p>\frac{N+2}{2}$, we have

$$\begin{aligned} \sigma :=\frac{p-1}{p}\left( 2+\frac{4}{N}\right) >2. \end{aligned}$$

Moreover,

$$\begin{aligned} \frac{\sigma p}{p-1}=2+\frac{4}{N}, \end{aligned}$$

implying

$$\begin{aligned} \Vert v_j\Vert _{L^{\frac{\sigma p}{p-1}}\left( Q_T\right) } \le C\Vert v_j\Vert _{W(0, T)}. \end{aligned}$$

We now can use Hölder’s inequality to estimate with (A.7)

A.12 $$\begin{aligned} \begin{aligned} \int _0^T \int _{\Omega } f^{\varepsilon }_i v_{j+1} \,d x d t&\le \int _0^T \int _{\Omega } f^{\varepsilon }_i v_{j+1}\left( \frac{2^{j+1}}{k}\right) ^{\sigma -1} v_j^{\sigma -1} \,d x d t \\ {}&\le \left( \frac{2^{j+1}}{k}\right) ^{\sigma -1} \int _0^T \int _{\Omega }\left| f^{\varepsilon }_i \Vert v_j\right| ^\sigma \,d x d t \\ {}&\le C\left( 2^{\sigma -1}\right) ^j\Vert f^{\varepsilon }_i\Vert _{L^p\left( Q_T\right) }\Vert v_j\Vert ^\sigma _{L^{\frac{\sigma p}{p-1}} \left( Q_T\right) } \\ {}&\le C\left( 2^{\sigma -1}\right) ^j\Vert f^{\varepsilon }_i\Vert _{L^p\left( Q_T\right) }\Vert v_j\Vert _{W(0, T)}^\sigma . \end{aligned} \end{aligned}$$

Inserting (A.10), (A.11) and (A.12) into (A.9) leads to

A.13 $$\begin{aligned} \Vert v_{j+1}\Vert _{W(0, T)}^2 \le C\left( 1+\Vert f^{\varepsilon }_i\Vert _{L^p\left( Q_T\right) }\right) B^j\left( \Vert v_j\Vert _{W(0, T)}^{2+\frac{4}{N}}+\Vert v_j\Vert _{W(0, T)}^\sigma \right) \nonumber \\ \end{aligned}$$

for all $j \ge 0$, where $B=\max \left\{ 2^{\frac{4 }{N}}, 2^{\sigma -1}\right\}$.

By setting $Y_j=\Vert v_j\Vert _{W(0, T)}^2$, we obtain a sequence $\left\{ Y_n\right\} _{n \ge 1}$ satisfying the property in Lemma A.2. It remains to show that $Y_1$ is small enough.

We show now that for any $\eta >0$, there exists $k \ge \max \left\{ 1, 2\Vert u^{\varepsilon }_0\Vert _{L^{\infty }(\Omega )}\right\}$ large enough such that

A.14 $$\begin{aligned} Y_1=\Vert v_1\Vert ^2_{W(0, T)} \le \eta . \end{aligned}$$

Let $p> 1$. By multiplying (2.1) by $p |u_i^{\varepsilon }|^{p-1}$ and integrating over $\Omega$, we obtain

A.15 $$\begin{aligned} \begin{aligned} \frac{d}{dt}\Vert u_i^{\varepsilon }\Vert ^{p}_{L^{p}(\Omega )} -p\int _{\Omega }\nabla \cdot (D(x,t) \Phi (u^{\varepsilon }) \nabla u_i^{\varepsilon }) |u_i^{\varepsilon }|^{p-1} \,dx =p\int _{\Omega }f_i^{\varepsilon } |u_i^{\varepsilon }|^{p-1} \,dx. \end{aligned} \end{aligned}$$

Integration by parts and the homogeneous Neumann boundary condition $(D(x,t) \Phi (u^{\varepsilon }) \nabla u_i^{\varepsilon })\cdot \nu = 0$ lead to

$$\begin{aligned} \begin{aligned} -p\int _{\Omega }&\nabla \cdot (D(x,t) \Phi (u^{\varepsilon }) \nabla u_i^{\varepsilon }) |u_i^{\varepsilon }|^{p-1}\,dx\\ {}&\ge \lambda M_ip(p-1)\int _{\Omega }|u_i^{\varepsilon }|^{b+p-2}|\nabla u_i^{\varepsilon }|^2 \,dx\\ {}&=\lambda M_ip(p-1)\frac{4}{(b+p)^2}\int _{\Omega }|\nabla \left( (u_i^{\varepsilon })^{\frac{b+p}{2}}\right) |^2 \,dx\\&=:C(p,\lambda , M_i)\int _{\Omega }|\nabla \left( (u_i^{\varepsilon })^{\frac{b+p}{2}}\right) |^2 \,dx, \end{aligned} \end{aligned}$$

where we used (1.3) and (Q2). By Hölder’s inequality

$$\begin{aligned} \left| p\int _{\Omega }f_i^{\varepsilon } |u_i^{\varepsilon }|^{p-1} \,dx\right| \le p\Vert f_i^{\varepsilon }\Vert _{L^{p}(\Omega )}\Vert u_i^{\varepsilon }\Vert _{L^{p}(\Omega )}^{p-1}. \end{aligned}$$

Therefore, it follows from (A.15) that

A.16 $$\begin{aligned} \frac{d}{dt}\Vert u_i^{\varepsilon }\Vert _{L^{p}(\Omega )}^{p} + C(p,\lambda , M_i) \int _{\Omega }\left| \nabla \left( (u_i^{\varepsilon })^{\frac{b+p}{2}}\right) \right| ^2dx \le p\Vert f_i^{\varepsilon }\Vert _{L^{p}(\Omega )}\Vert u_i^{\varepsilon }\Vert _{L^{p}(\Omega )}^{p-1}. \end{aligned}$$

By applying for $r<1$ the elementary inequality

$$\begin{aligned} y' \le \alpha (t)y^{1-r} \quad \Longrightarrow \quad y(T) \le \left[ y(0)^{r}+ r \int _0^T\alpha (t) \,dt\right] ^{1/r}, \end{aligned}$$

to (A.16) with $r=1/p$ and $y(t) = \Vert u_i^{\varepsilon }(t)\Vert _{L^{p}(\Omega )}^{p}$, we obtain

A.17 $$\begin{aligned} \begin{aligned} \Vert u_i^{\varepsilon }(T)\Vert _{L^{p}(\Omega )}^{p}&\le \left[ \Vert u^{\varepsilon }_0\Vert _{L^{p}(\Omega )} + \int _0^{T}\Vert f_i^{\varepsilon }\Vert _{L^{p}(\Omega )} \,dt \right] ^{ p} \\ {}&\le \left[ \Vert u^{\varepsilon }_0\Vert _{L^{p}(\Omega )} + \Vert f_i^{\varepsilon }\Vert _{L^{p}(Q_T)}T^{\frac{(p-1)}{p}} \right] ^{p} =: C_{T}. \end{aligned} \end{aligned}$$

That means

A.18 $$\begin{aligned} u_i^{\varepsilon } \in L^{\infty }(0,T;L^{p}(\Omega )) \quad \text { and } \quad \Vert u_i^{\varepsilon }(T)\Vert _{L^{p}(\Omega )}^{p} \le C_{T} \end{aligned}$$

where $C_{T}$ defined in (A.17) grows at most polynomially in *T*. By integrating (A.16) with respect to *t* on (0, *T*) and by using Young’s inequality and the convention $r:= b+p\ge p>1$, we get

$$\begin{aligned} \begin{aligned} C(p,\lambda , M_i)\int ^T_0\int _{\Omega }\left| \nabla \left( (u_i^{\varepsilon })^{\frac{r}{2}}\right) \right| ^2 \,dxdt&\le \Vert u_0^{\varepsilon }\Vert _{L^{p}(\Omega )}^{p} + p\int _0^T\Vert f_i^{\varepsilon }\Vert _{L^{p}(\Omega )}\Vert u_i^{\varepsilon }\Vert _{L^{p}(\Omega )}^{p-1} \,dt\\&\le \Vert u_0^{\varepsilon }\Vert _{L^{p}(\Omega )}^{p} + p\Vert f_i^{\varepsilon }\Vert _{L^{p}(Q_T)}\Vert u_i^{\varepsilon }\Vert _{L^{p}(Q_T)}^{p-1}. \end{aligned} \end{aligned}$$

By adding $C(p,\lambda , M_i)\int ^T_0\int _{\Omega }(u_i^{\varepsilon })^{r} \,d x d t$ to both sides, we have

A.19 $$\begin{aligned} \begin{aligned} C(p,\lambda , M_i)&\int _0^T\Vert (u_i^{\varepsilon })^{\frac{r}{2}}\Vert _{H^1(\Omega )}^2 \,dt\\ {}&= C(p,\lambda , M_i)\int _0^T\left[ \int _{\Omega }\left| \nabla \left( (u_i^{\varepsilon })^{\frac{r}{2}}\right) \right| ^2 \,dx + \int _{\Omega }\left| (u_i^{\varepsilon })^{\frac{r}{2}} \right| ^2 \,dx\right] dt\\ {}&\le \Vert u_0^{\varepsilon }\Vert _{L^{p}(\Omega )}^{p} + p\Vert f_i^{\varepsilon }\Vert _{L^{p}(Q_T)}\Vert u_i^{\varepsilon }\Vert _{L^{p}(Q_T)}^{p-1} + C(p,\lambda , M_i)\int _0^T\Vert u_i^{\varepsilon }\Vert _{L^{r}(\Omega )}^{r} \,dt. \end{aligned} \end{aligned}$$

By the Sobolev’s embedding, we have

A.20 $$\begin{aligned} C(p,\lambda , M_i)\int _0^T\Vert (u_i^{\varepsilon })^{\frac{r}{2}}\Vert ^2_{H^1(\Omega )}\,dt \ge C(p,\lambda , M_i) \,C_S^2\int _0^T \Vert u_i^{\varepsilon }\Vert _{L^{s}(\Omega )}^{r} \,dt \nonumber \\ \end{aligned}$$

with

$$\begin{aligned} s = {\left\{ \begin{array}{ll} \frac{r N}{N-2} &{}\text { if } N \ge 3,\\ r< s < \infty \text { arbitrary } &{}\text { if } N = 1,2. \end{array}\right. } \end{aligned}$$

On the other hand, by using the bound $\Vert u_i^{\varepsilon }(t)\Vert _{L^{p}(\Omega )}^{p} \le C_{T}$ in (A.18) and the interpolation inequality

$$\begin{aligned} \Vert u_i^{\varepsilon }\Vert _{L^{r}(\Omega )} \le \Vert u_i^{\varepsilon }\Vert _{L^{p}(\Omega )}^{\alpha }\Vert u_i^{\varepsilon }\Vert _{L^{s}(\Omega )}^{1-\alpha } \le C_{T}^{\frac{\alpha }{p}}\Vert u_i^{\varepsilon }\Vert _{L^{s}(\Omega )}^{1-\alpha } \end{aligned}$$

with

$$\begin{aligned} \frac{1}{r} = \frac{\alpha }{p} + \frac{1-\alpha }{s} \quad \text {and} \quad \alpha = \frac{2p}{2p+bN}\in (0,1], \end{aligned}$$

we estimate in the cases $b>0$ for which $\alpha <1$

A.21 $$\begin{aligned} \begin{aligned} C(p,\lambda , M_i)\int _0^T\Vert u_i^{\varepsilon }\Vert _{L^{r}(\Omega )}^{r} \,dt&\le C(p,\lambda , M_i )\int _{0}^{T}C_{T}^{\frac{\alpha r}{p}} \Vert u_i^{\varepsilon }\Vert _{L^{s}(\Omega )}^{(1-\alpha )r} \,dt \\ {}&\le \frac{C(p,\lambda , M_i)\,C_S^2}{2}\int _0^T\Vert u_i^{\varepsilon }\Vert _{L^{s}(\Omega )}^{r} \,dt + CC_{T}^{\frac{r}{p}}T, \end{aligned}\nonumber \\ \end{aligned}$$

where we have used Young’s inequality (with the exponents $1=(1-\alpha ) + \alpha$) in the last step. Note that if $b=0$, the bound (A.21) still holds true without the first term and with $\frac{r}{p}=1$. Inserting (A.20) and (A.21) into (A.19) leads to

A.22 $$\begin{aligned} \begin{aligned} \int _0^T\Vert u_i^{\varepsilon }\Vert _{L^{s}(\Omega )}^{r}dt&\le \frac{2}{C(p,\lambda , M_i)\,C_S^2}\left[ \Vert u_0^{\varepsilon }\Vert _{L^{p}(\Omega )}^{p} + p \Vert f_i^{\varepsilon }\Vert _{L^{p}(Q_T)}\Vert u_i^{\varepsilon }\Vert _{L^{p}(Q_T)}^{p-1} + CC_{T,0}^{\frac{r}{p}}T\right] \\ {}&\le \frac{2}{C(p,\lambda , M_i)\,C_S^2}\left[ \Vert u_0^{\varepsilon }\Vert _{L^{p}(\Omega )}^{p} + p\Vert f_i^{\varepsilon }\Vert _{L^{p}(Q_T)} C_{T}^{\frac{p-1}{p}} + CC_{T}^{\frac{r}{p}}T\right] \\ {}&=: D_{T} \quad (\text {use} (A.18)). \end{aligned} \end{aligned}$$

It follows that

$$\begin{aligned} u^{\varepsilon }_i \in L^{r}(0,T;L^{s}(\Omega )) \end{aligned}$$

with

$$\begin{aligned} {\left\{ \begin{array}{ll} s = \frac{r N}{N-2} &{}\text { if } N\ge 3,\\ r< s <\infty \text { arbitrary } &{}\text { if } N=1,2 \end{array}\right. } \end{aligned}$$

and

$$\begin{aligned} \int _0^T\Vert u_i^{\varepsilon }\Vert _{L^{s}(\Omega )}^{r} \,dt \le D_{T} \end{aligned}$$

with $D_{T}$ defined in (A.22).

Thus, we have

$$\begin{aligned} \Vert u^{\varepsilon }_i\Vert _{L^{\infty }\left( 0, T; L^p(\Omega )\right) }+\Vert u^{\varepsilon }_i\Vert _{L^{r}\left( 0, T; L^s(\Omega )\right) } \le C_T, \end{aligned}$$

where $r=b+p \ge p$ and $s=\frac{r N}{N-2}$ if $N \ge 3$ and $r<s<+\infty$ arbitrary if $N \le 2$.

Using Lemma A.1, we have

$$\begin{aligned} \Vert u^{\varepsilon }_i\Vert _{L^{\tau }\left( Q_T\right) } \le C_T \quad \text{ with } \quad \tau = {\left\{ \begin{array}{ll} \frac{N r +2 p}{N} &{} \text{ if } N \ge 3, \\ <r +p \text{ arbitrary } &{} \text{ if } N \le 2. \end{array}\right. } \end{aligned}$$

Direct calculations show that $\tau >2+\frac{4}{N}$ if $N \ge 2$ and $\tau >3$ if $N=1$. In particular,

A.23 $$\begin{aligned} \Vert u^{\varepsilon }_i\Vert _{L^{2+\frac{4}{N}}\left( Q_T\right) } \le C_T \text{ for } d \ge 2 \text{ and } \Vert u^{\varepsilon }_i\Vert _{L^3\left( Q_T\right) } \le C_T \text{ for } d=1. \end{aligned}$$

From (A.9),

A.24 $$\begin{aligned} \Vert v_1\Vert _{W(0, T)}^2 \le C\left( \int _0^T\Vert v_1(t)\Vert ^2_{L^{2}(\Omega )} \,d t+\Vert v_1(0)\Vert ^2_{L^{2}(\Omega )} + \int _0^T \int _{\Omega } f^{\varepsilon }_i v_1 \,dxdt\right) . \end{aligned}$$

Since $$k \ge 2\Vert u^{\varepsilon }_0\Vert _{L^{\infty }(\Omega )},\Vert v_1(0)\Vert ^2_{L^{2}(\Omega )}=\Vert \left( u^{\varepsilon }_0-\frac{k}{2}\right) _+\Vert ^2_{L^{2}(\Omega )}=0$$.

Consider now the case $N \ge 2$. By using (A.7), it yields

A.25 $$\begin{aligned} \begin{aligned} \int _0^T \int _{\Omega }\left| v_1\right| ^2 \,d x d t&=\int _0^T \int _{\Omega } \textbf{1}_{A_1}\left| v_1\right| ^2 \,d x d t \le \left( \frac{4}{k}\right) ^{\frac{4}{N}} \int _0^T \int _{\Omega }\left| v_0\right| ^{2+\frac{4}{N}} \,d x d t \\ {}&\le \left( \frac{4}{k}\right) ^{\frac{4}{N}}\Vert u^{\varepsilon }_i\Vert _{L^{2+\frac{4}{N}}(Q_T)}^{2+\frac{4}{N}} \le \left( \frac{4}{k}\right) ^{\frac{4}{N}} C_T, \end{aligned} \end{aligned}$$

recalling that $v_0=(u^{\varepsilon }_i)_{+}$. Similarly to (A.12), we get

A.26 $$\begin{aligned} \int _0^T \int _{\Omega } f^{\varepsilon }_i v_1 \,d x d t \le \left( \frac{4}{k}\right) ^{\sigma -1}\Vert f^{\varepsilon }_i\Vert _{L^p\left( Q_T\right) }\Vert u^{\varepsilon }_i \Vert _{L^{2+\frac{4}{N}}\left( Q_T\right) }^\sigma \le \left( \frac{4}{k}\right) ^{\sigma -1} C_T. \end{aligned}$$

From (A.24), (A.25) and (A.26), we get (A.14) if

$$\begin{aligned} k=4 \max \left\{ \left( \frac{C_T}{\eta }\right) ^{\frac{N}{4}}, \left( \frac{C_T}{\eta }\right) ^{\frac{1}{\sigma -1}}\right\} . \end{aligned}$$

Thus, with this choice of *k*, it follows that

$$\begin{aligned} 0=\lim _{j \rightarrow \infty } Y_j=\Vert (u^{\varepsilon }_i-k)_{+}\Vert ^2 \end{aligned}$$

and hence,

$$\begin{aligned} \Vert u^{\varepsilon }_i\Vert _{L^{\infty }\left( Q_T\right) } \le k=4 \max \left\{ \left( \frac{C_T}{\eta }\right) ^{\frac{N}{4}}, \left( \frac{C_T}{\eta }\right) ^{\frac{1}{\sigma -1}}\right\} \end{aligned}$$

which is our desired estimate.

The proof for the case $N=1$ is very similar using

$$\begin{aligned} \int _0^T \int _{\Omega }\left| v_1\right| ^2 \,d x d t \le \frac{4}{k} \int _0^T \int _{\Omega }\left| v_0\right| ^3 \,d x d t \le \frac{4}{k} C_T \end{aligned}$$

and

$$\begin{aligned} \int _0^T \int _{\Omega } f^{\varepsilon }_i v_1 \,d x d t \le \left( \frac{4}{k}\right) ^{\frac{4 \xi }{1+2 \xi }}\Vert f^{\varepsilon }_i\Vert _{L^p\left( Q_T\right) }\Vert u^{\varepsilon }_i\Vert _{L^3(Q_T)}^{1+\frac{4 \xi }{1+2 \xi }} \le \left( \frac{4}{k}\right) ^{\frac{4 \xi }{1+2 \xi }} C_T \end{aligned}$$

where $\xi =\frac{1}{2}(2p-3)>0$. We therefore omit the details.

Thus, we completed the proof of Lemma 2.9. $\square$

## Footnotes

1 The domain $\Omega$ was considered in $\mathbb {R}^2$ and $\mathbb {R}^3$ in [31] and [1], respectively. For the current work, we can deal with the case of arbitrary dimension.

2 In fact, [1] generalised the diffusion rate to $\kappa (n)$ for a continuous function $\kappa (\cdot )$, but it does not alter the fact that the diffusion rates must be the same so that the technique therein can be applied.

3 In fact, thanks to the lower bound of *n*, it was possible to replace all diffusion operators by a common function $\kappa (n)$, where $\kappa : (0,\infty )\rightarrow (0,\infty )$ is a continuous function.
